# Lymphocytic Choriomeningitis Virus Seroprevalence in a Cohort of German Forestry Workers

**DOI:** 10.3390/v18010004

**Published:** 2025-12-19

**Authors:** Calvin Mehl, Jonas Schmidt-Chanasit, Beate Becker-Ziaja, Sandra Werdermann, Olaf Niederstraßer, Merle M. Böhmer, Rainer G. Ulrich

**Affiliations:** 1 Institute of Infectology, Friedrich-Loeffler-Institut (FLI), Südufer 10, 17493 Greifswald–Insel Riems, Germany; calvin.mehl@fli.de; 2 Department of Arbovirology and Entomology, Bernhard Nocht Institute for Tropical Medicine, WHO Collaborating Centre for Arbovirus and Haemorrhagic Fever Reference and Research, Bernhard-Nocht-Strasse 74, 20359 Hamburg, Germany; jonassi@gmx.de; 3 Faculty of Mathematics, Informatics and Natural Sciences, University of Hamburg, Ohnhorststrasse 18, 22609 Hamburg, Germany; 4 Department of Virology, Bernhard Nocht Institute for Tropical Medicine, Bernhard-Nocht-Strasse 74, 20359 Hamburg, Germany; beate.becker-ziaja@bnitm.de; 5 Institut für Arbeits- und Sozialhygiene Stiftung, Perleberger Str. 31, 16866 Kyritz, Germany; s.werdermann@asw-kyritz.de; 6 Helios Kliniken, Pieskower Straße 33, 15526 Bad Saarow, Germany; olaf.niederstrasser@bergmannstrost.de; 7 Department for Infectious Disease Epidemiology, Bavarian Health and Food Safety Authority (LGL), Ridlerstr. 75, 80339 Munich, Germany; merle.boehmer@lgl.bayern.de; 8 Institute of Social Medicine and Health Systems Research, Otto-von-Guericke-University, Leipziger Str. 44, 39120 Magdeburg, Germany; 9 Institute of Novel and Emerging Infectious Diseases, Friedrich-Loeffler-Institut (FLI), Südufer 10, 17493 Greifswald–Insel Riems, Germany

**Keywords:** LCMV, Brandenburg, north-east Germany, antibodies, zoonosis, house mice

## Abstract

Forestry workers are exposed, through their occupation, to a variety of zoonotic pathogens. Lymphocytic choriomeningitis virus (LCMV) is a zoonotic agent typically transmitted through the excreta of infected rodents. Current knowledge concerning the prevalence of LCMV in wild house mice (*Mus musculus*) in Germany is limited, with the majority of data coming from studies during the 1960s and 1970s and only from the western and southern federal states. In this study, blood samples from 563 forestry workers, collected in 2008 from ten forestry offices in Brandenburg, Germany, were screened for LCMV-reactive antibodies. In total, LCMV-reactive antibodies were detected in 1.4% (8/563) of samples. The seroprevalence varied between 0% and 6.3% depending on the forestry office, with the highest prevalence in Alt Ruppin. A parallel serological pilot study of house mice from a neighbouring federal state also indicates a very low prevalence. Although forestry workers are often at increased risk of zoonotic infection, this seroprevalence is comparable to that from a 1960s study from what was, at that time, West Germany. This study provides the first evidence of LCMV in humans from Brandenburg and highlights the need for an increased LCMV screening effort in humans and wild rodents in Germany.

## 1. Introduction

Lymphocytic choriomeningitis virus (LCMV, species *Mammarenavirus choriomeningitidis*) is a globally distributed zoonotic pathogen primarily spread by house mice (*Mus musculus*) [1]. The virus is typically transmitted to humans through contact with the excreta or secreta of infected rodents [2,3]. LCMV infections may be severely underdiagnosed because they are often asymptomatic or present with non-specific self-limiting flu-like illness, including headache, fever, myalgia, malaise, nausea, and vomiting [4,5]. While more severe courses of disease can cause meningoencephalitis [1], the aetiological agent responsible for developing encephalitis is not identified in more than half of cases [6,7]. An LCMV infection may be particularly lethal for immunocompromised individuals, such as solid organ transplant patients [8] and those living with human immunodeficiency virus (HIV) [9]. LCMV is also a severe teratogenic agent, causing birth defects including hydrocephaly, microcephaly, and retinal damage [10,11,12].

In Germany, LCMV is not a notifiable disease, but it has been detected in humans, wild house mice, and pet Syrian golden hamsters (*Mesocricetus auratus*) since the 1960s (reviewed in [13]). A 1964 study in what was, at that time, West Germany, provides the most comprehensive screening of LCMV in wild house mice [14]. This study found the highest prevalence in the western-most federal state of North Rhine-Westphalia [14], the same federal state where the first outbreak of callitrichid hepatitis (CH), a lethal disease in New World primates caused by LCMV, occurred in Germany [15]. In the 1960s and 1970s, human LCMV infections were reported across West Germany, with most cases occurring in North Rhine-Westphalia and Hesse, with pet Syrian golden hamsters implicated in the transmission [1,11,16,17]. In these cases, the virus was detected only because the patients suffered from meningitis or prenatal infections. LCMV later re-emerged in Germany with an outbreak of CH in a zoo in Hesse, where wild house mice were implicated in the transmission [18]. A novel lineage of LCMV was also detected in wild wood mice (*Apodemus sylvaticus*) in the southern federal state of Bavaria [19]. In addition, a study along the house mouse hybridization zone detected LCMV in the Czech Republic, but not in the German state of Bavaria [20]. However, these studies represent only the western and southern federal states of Germany, and almost nothing is known on the occurrence of LCMV in eastern Germany.

Here, forestry workers from ten forest districts in Brandenburg in north-east Germany were screened for LCMV-reactive antibodies. These sera have previously been investigated for reactive antibodies against hantaviruses (Puumala virus, Tula virus, and Dobrava–Belgrade virus) [21], *Rickettsia* spp. [22], hepeviruses, i.e., hepatitis E virus (*Paslahepevirus balayani*) and rat hepatitis E virus (*Rocahepevirus ratti*) [23], and *Toxoplasma gondii* [24,25]. In parallel, a pilot serological study of house mice from eastern, northern and western Germany was performed.

## 2. Materials and Methods

Forestry workers from ten forest districts in Brandenburg were invited by the Federal Ministry of Brandenburg to participate in this study. Between May and June 2008, 563 participants provided blood samples and filled out a standardized questionnaire. Participants provided written informed consent prior to completing the questionnaire and providing a blood sample. Blood samples were stored at −20 °C until being screened for LCMV-reactive antibodies at the Bernhard Nocht Institute for Tropical Medicine (BNITM, Hamburg).

House mice were collected by pest controllers in North Rhine-Westphalia, Baden-Wuerttemberg, Schleswig-Holstein, and Saxony-Anhalt [26,27,28,29]. Chest cavity fluid (CCF) was collected during dissection by rinsing the chest cavity using 1 mL phosphate-buffered saline (PBS). In addition, the pericardium was removed, from which the blood for the immunofluorescence assay (IFA) could then be obtained.

IFA for LCMV immunoglobulin G (IgG) detection in humans was performed with LCMV Armstrong strain-infected Vero E6 cells, as described previously [30]. In brief, Vero E6 cells were spread onto slides, air dried, and fixed in acetone. Human serum samples were serially diluted in PBS, starting with an initial dilution of 1:10, which was added to the cells and incubated for 90 min at 37 °C. After washing with PBS, slides were incubated with fluorescein isothiocyanate (FITC)-labelled rabbit anti-human IgG antibodies (SIFIN, Berlin, Germany) at 37 °C for 25 min. IgG titers of 1:20 or more were considered positive.

For house mice, this test was slightly modified by using Mopeia virus-/LCMV-infected Vero E6 cells to screen the undiluted CCF samples. FITC-labelled goat anti-mouse IgG antibodies (Invitrogen), diluted 1:500, were used as secondary antibodies. For confirmation, the corresponding pericardial blood was then tested undiluted and diluted 2-fold to 1:16 separately on LCMV- and Mopeia virus-infected Vero E6 cells.

The 95% confidence interval (CI) was calculated in RStudio 2025.05.01+513 [31].

## 3. Results

**Figure 1:**
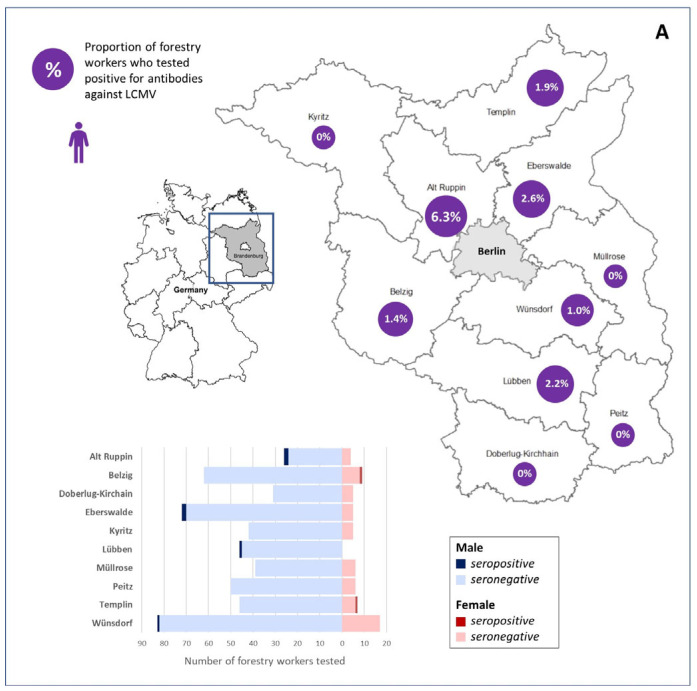

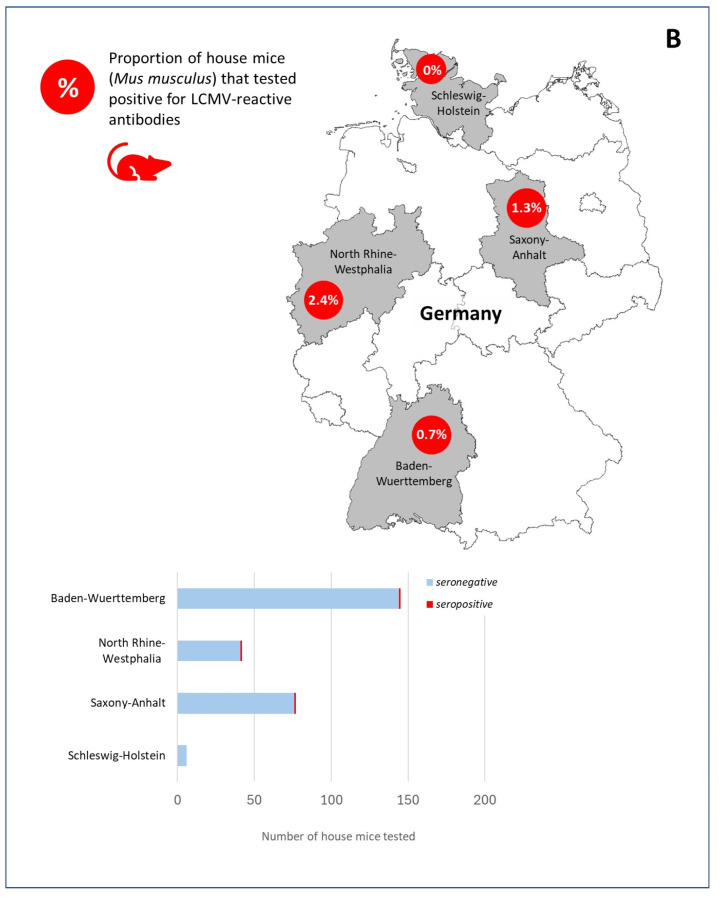
Map and distribution of LCMV seroprevalence in forestry workers from 10 forestry offices in Brandenburg, Germany (**A**), and in house mice from Schleswig-Holstein (isle of Helgoland), North Rhine-Westphalia (Münster, Cologne, Dortmund, and other sites), Baden-Wuerttemberg (Stuttgart), and Saxony-Anhalt (Magdeburg) (**B**). Map data was kindly provided by Landesbetrieb Forst Brandenburg.

Of the 563 forestry workers in this study, 8 (1.4%; 95% CI 0.6–2.8) had LCMV-reactive IgG antibodies (Table 1 and Table S1). Forestry workers were predominantly male (88.6%), though IgG antibodies were detected in both males and females, with no significant difference in seroprevalence observed between the sexes (Table 1). The small difference in the average age at the different forestry offices and the low prevalence observed hampered a regression analysis for the potential age influence on the seroprevalence. The highest IgG seroprevalence was detected in Alt Ruppin (6.3%; 95% CI 0.8–20.8; Table 1 and Figure 1A).

In parallel, we investigated a total of 270 house mice from eastern Germany (Magdeburg, Saxony-Anhalt), northern Germany (isle of Helgoland, Schleswig-Holstein) and western Germany (North Rhine-Westphalia; Baden-Wuerttemberg). Single seropositive house mice were detected in Magdeburg (1/77), Stuttgart (1/145), and Dortmund (1/4) (Figure 1B; Table S2). All animals with positive CCF samples also tested positive in the pericardial blood samples.

## 4. Discussion and Conclusions

IgG antibodies against LCMV were detected in 1.4% of forestry workers from Brandenburg, Germany. The IgG seroprevalence varied by forest district, with a prevalence between 0% and 6.3%. The only seroprevalence data for comparison from Germany comes from the period 1962 to 1964 and only includes patients from what was, at that time, West Germany [17]. In the respective study, the authors found a seroprevalence of 4.1% and found no significant difference between the sexes and no difference between farm workers and other professions [17]. However, the authors do note a significantly higher seroprevalence in regions where LCMV had previously been detected in wild house mice [17]. More recently, a retrospective study of patients from Austria with suspected meningoencephalitis detected only a single LCMV-RNA-positive sample from a cohort of nearly 400 [32]. In a study of wild rodents from Austria, a single wood mouse tested positive for anti-LCMV antibodies [33]. Here, we also investigated house mice from Magdeburg, Saxony-Anhalt, neighbouring the federal state of Brandenburg. In only one of the 77 CCF and pericardial blood samples did we detect LCMV-reactive antibodies. A similar low seroprevalence was seen in house mice from Stuttgart (1/145) and North Rhine-Westphalia (1/42); however, in Dortmund, a city in which a callitrichid hepatitis outbreak in New World primates has previously been reported [15], one of four house mice tested seropositive. Together, these studies indicate, in general, a low prevalence of LCMV in humans and house mice in western Europe and highlight the need for new screening in these and other parts of Germany and in the general human population, as well as in house mice, wood mice, and other potential reservoirs.

The European house mouse hybrid zone (HMHZ) is a region approximately 20 km wide that stretches from Scandinavia to the Black sea, where two subspecies of house mice (*Mus musculus domesticus* [Mmd] to the West and *Mus musculus musculus* [Mmm] to the East) meet and hybridize [34]. This hybrid zone passes through Brandenburg from North to South, on the Eastern side of Berlin [35], and acts as a natural barrier to endoparasite transmission between subspecies [35,36,37]. A similar phenomenon was recently reported for Tula hantavirus (TULV) in a hybrid zone of common vole (*Microtus arvalis*) lineages, which are the main reservoir of TULV. A strong association between the distribution of two major phylogenetic clades of TULV and the rodent host lineages in this natural hybrid zone of the European common vole suggests a strong barrier for effective virus transmission despite frequent dispersal and gene flow among local host populations [38]. In contrast, the observation made in a zoo in Frankfurt, i.e., the co-occurrence of LCMV lineages I and II in *Mus musculus domesticus,* must be caused, to our current knowledge, by an artificial introduction of lineage II LCMV in a region where only lineage I would be expected [39]. According to the proposed host–phylogeographic relationship between house mouse subspecies and LCMV genetic lineages, such that LCMV lineage I occurs in Mmd and LCMV lineage II occurs in Mmm [20], two lineages of LCMV may exist in Brandenburg, with potential limitations in transmission between the house mouse subspecies and its hybrids. Therefore, forestry workers in this region may be exposed in residential areas by both lineages I and II.

However, forestry workers may also be exposed to LCMV through contact with wood mice. Wood mice, being habitat generalists, can be found in both forested and agricultural habitats [40,41]. Although these mice generally prefer habitats with high seed abundance, such as forests and conifer plantations [42], they can also be found in high densities in croplands [41]. This species was recently associated with a novel lineage of LCMV in Germany [19]. The association between wood mice and forested areas may increase the risk of occupational exposure for forestry workers. However, as mentioned above, forestry workers might be further affected by exposure to house mouse habitats during leisure activities and in residential areas.

Although the seroprevalence of LCMV in forestry workers, here, cannot be compared to that of the general public, because of the absence of data, we do know that this profession is often associated with increased risk of zoonotic exposure. Recreational activities and occupations that require extended periods in forests, or in close contact with wild animals or their excreta, such as hunting and working in forestry, respectively, may increase the risk of zoonotic pathogen transmission. In Europe, hunters and forestry workers are often exposed to wild rodents and their excreta, increasing their risk of rodent-borne zoonotic infections [43]. Hunters and forestry workers are exposed to a diverse range of zoonotic pathogens and parasites, including *Francisella tularensis* [44,45,46], hepatitis E virus [47,48], *Borrelia burgdorferi*, hantaviruses, *Echinococcus* spp., *Anaplasma phagocytophilum* [48,49,50], *Coxiella burnetii*, *Rickettsia* spp. [51,52], *Leptospira* spp. [46], *Bartonella* spp., *Babesia microti*, *Toxocara canis*, *Trichinella spiralis*, and *Echinococcus granulosus* [50].

Because nothing is known on the prevalence and genetic diversity of LCMV in rodents from this region, and antibodies that are reactive against different arenavirus species and LCMV lineages are often cross-reactive [53,54], we cannot fully exclude infection by other arenavirus species or identify the LCMV lineage (I, II, or V) causing the seroconversion in the forestry workers in this study. However, being the only zoonotic arenavirus reported in Germany, LCMV is the most likely arenavirus in these individuals. This study provides the first evidence for the circulation of LCMV in Brandenburg, based on sera collected in 2008, and highlights the need for broader and more current RNA and antibody screening in humans, wild house mice, wood mice, and other potential reservoirs in Brandenburg and other parts of Germany. Although this data provides novel insight into the presence of LCMV in previously unrepresented regions of Germany, namely in eastern Germany, in the future an updated screening of house mice, wood mice, and potential other reservoirs is needed to understand the current pathogen circulation. In addition, the seroprevalence and driving epidemiological factors needs to be comparatively investigated for the forestry worker cohort from Brandenburg, including previously published results for hantaviruses [21], hepeviruses [23], *Rickettsia* spp. [22], *Toxoplasma gondii* [24,25] and the additional viral and bacterial pathogens investigated. Finally, this pilot study in Brandenburg aims to increase the awareness of physicians of this neglected pathogen.

## Figures and Tables

**Table 1:** LCMV seroprevalence in forestry workers from Brandenburg, Germany.

Forestry Office	Total Number	Sex	Number (%)	Average Age ± Standard Deviation (Years)	Number Anti-LCMV-IgG Positive (%) and 95% CI	Number Anti-LCMV-IgG Positive Total (%) and 95% CI
Alt Ruppin	32	Male	28 (87.5)	48.54 ± 6.68	2 (7.1) 0.9–23.5	2 (6.3) 0.8–20.8
		Female	4 (12.5)	47.25 ± 9.03	0 (0) 0.0–60.2	
Doberlug-Kirchhain	36	Male	31 (86.1)	52.29 ± 4.87	0 (0) 0.0–11.2	0 (0) 0.0–9.7
		Female	5 (13.9)	48.20 ± 5.63	0 (0) 0.0–52.2	
Belzig	71	Male	62 (87.3)	49.23 ± 8.30	0 (0) 0.0–5.8	1 (1.4) 0.0–7.6
		Female	9 (12.7)	51.00 ± 3.97	1 (11.1) 0.3–48.2	
Wünsdorf	101	Male	83 (82.2)	20 ± 7.49	1 (1.2) 0.0–6.5	1 (1.0) 0.0–5.4
		Female	19 (17.8)	46.17 ± 6.54	0 (0) 0.0–18.5	
Lübben	46	Male	46 (100.0)	49.37 ± 6.48	1 (2.2) 0.1–11.5	1 (2.2) 0.1–11.5
		Female	0 (0.0)	NA *	0 (0) NA *	
Kyritz	47	Male	42 (89.4)	49.05 ± 6.68	0 (0) 0.0–8.4	0 (0) 0.0–7.5
		Female	5 (10.6)	49.60 ± 6.39	0 (0) 0.0–52.2	
Peitz	56	Male	50 (89.3)	48.68 ± 7.90	0 (0) 0.0–7.1	0 (0) 0.0–6.4
		Female	6 (10.7)	42.67 ± 6.31	0 (0) 0.0–45.9	
Eberswalde	77	Male	72 (93.5)	45.93 ± 7.35	2 (2.8) 0.3–9.7	2 (2.6) 0.3–9.1
		Female	5 (6.5)	51.40 ± 3.36	0 (0) 0.0–52.2	
Templin	52	Male	46 (88.5)	44.00 ± 8.35	0 (0) 0.0–7.7	1 (1.9) 0.0–10.3
		Female	6 (11.5)	45.67 ± 11.43	1 (16.7) 0.0–64.1	
Müllrose	45	Male	39 (86.7)	47.18 ± 8.13	0 (0) 0.0–9.0	0 (0) 0.0–7.9
		Female	6 (13.3)	54.50 ± 3.39	0 (0) 0.0–45.9	
**Total**	**563**	**Male**	**499 (88.6)**	**48.03 ± 7.62**	**6 (1.2)** **0.4–2.6**	**8 (1.4)** **0.6–2.8**
		**Female**	**64 (11.4)**	**48.08 ± 6.92**	**2 (3.1)** **0.4–10.8**	

* Not applicable, CI = confidence interval, total numbers are labeled in bold.

## Data Availability

All the data in this study are included in the manuscript and the Supplementary Tables S1 and S2.

## Supplementary Materials

The following Supplementary Material is available online at https://www.mdpi.com/article/10.3390/v18010004/s1, Table S1: Results of the investigation of forestry worker sera, Table S2: Detection of LCMV-reactive antibodies in house mice from different regions in Germany.

## Abbreviations

The following abbreviations are used in this manuscript:

LCMV	lymphocytic choriomeningitis virus
HMHZ	house mouse hybrid zone
IFA	immunofluorescence assay
IgG	immunoglobulin G
PBS	phosphate-buffered saline
CCF	chest cavity fluid
