# Supplementary material for: Lymphocytic Choriomeningitis Virus Seroprevalence in a Cohort of German Forestry Workers
Source: Viruses. 2025 Dec 19;18(1):4. doi: 10.3390/v18010004 (PMC12846623; doi:10.3390/v18010004)
Supplement: Supplementary file 1 [file viruses-18-00004-s001.zip › viruses-3968464-supplementary.pdf]

Lymphocytic Choriomeningitis Virus Seroprevalence in a Cohort of German Forestry Workers

Calvin Mehl 1, Jonas Schmidt-Chanasit 2,3, Beate Becker-Ziaja 4, Sandra Werdermann 5,†, Olaf Niederstraßer 6,‡, Merle M. Böhmer 7,8 and Rainer G. Ulrich 9,*

1 Institute of Infectology, Friedrich-Loeffler-Institut (FLI), Südufer 10, 17493 Greifswald–Insel Riems, Germany; calvin.mehl@fli.de
2 Department of Arbovirology and Entomology, Bernhard Nocht Institute for Tropical Medicine, WHO Collaborating Centre for Arbovirus and Haemorrhagic Fever Reference and Research, Bern-hard-Nocht-Strasse 74, 20359 Hamburg, Germany; jonassi@gmx.de
3 Faculty of Mathematics, Informatics and Natural Sciences, University of Hamburg, Ohnhorststrasse 18, 22609 Hamburg, Germany
4 Department of Virology, Bernhard Nocht Institute for Tropical Medicine, Bernhard-Nocht-Strasse 74, 20359 Hamburg, Germany; beate.becker-ziaja@bnitm.de
5 Institut für Arbeits- und Sozialhygiene Stiftung, Perleberger Str. 31, 16866 Kyritz, Germany; s.werdermann@asw-kyritz.de
6 Helios Kliniken, Pieskower Straße 33, 15526 Bad Saarow, Germany; olaf.niederstrasser@bergmannstrost.de
7 Department for Infectious Disease Epidemiology, Bavarian Health and Food Safety Authority (LGL), Ridlerstr. 75, 80339 Munich, Germany; merle.boehmer@lgl.bayern.de
8 Institute of Social Medicine and Health Systems Research, Otto-von-Guericke-University, Leipziger Str. 44, 39120 Magdeburg, Germany
9 Institute of Novel and Emerging Infectious Diseases, Friedrich-Loeffler-Institut (FLI), Südufer 10, 17493 Greifswald–Insel Riems, Germany
* Correspondence: rainer.ulrich@fli.de
† Current address: ASW Kyritz, Perleberger Straße 4, 16866 Kyritz, Germany.
‡ Current address: Klinik für Neurologie und Frührehabilitation, BG Klinikum Bergmannstrost Halle, Merseburger Str. 165, 06112 Halle/Saale, Germany.

SUPPLEMENT

Table S1: Results of the investigation of forestry worker sera.

ID	Patient	Forestry_office	Sampling_date	LCMV
1	1	Alt Ruppin	2008/05/19	Neg
2	2	Alt Ruppin	2008/05/19	Neg
3	3	Alt Ruppin	2008/05/19	Pos
4	4	Alt Ruppin	2008/05/19	Neg
5	5	Alt Ruppin	2008/05/19	Pos
6	6	Alt Ruppin	2008/05/19	Neg
7	7	Alt Ruppin	2008/05/19	Neg
8	8	Alt Ruppin	2008/05/19	Neg
9	9	Alt Ruppin	2008/05/19	Neg
10	10	Alt Ruppin	2008/05/19	Neg
11	11	Alt Ruppin	2008/05/19	Neg
12	12	Alt Ruppin	2008/05/19	Neg
13	13	Alt Ruppin	2008/05/19	Neg
14	14	Alt Ruppin	2008/05/19	Neg
15	15	Alt Ruppin	2008/05/19	Neg
16	16	Alt Ruppin	2008/05/19	Neg
17	17	Alt Ruppin	2008/05/19	Neg
18	18	Alt Ruppin	2008/05/19	Neg
19	19	Alt Ruppin	2008/05/19	Neg
20	20	Alt Ruppin	2008/05/19	Neg
21	21	Alt Ruppin	2008/05/19	Neg
22	22	Alt Ruppin	2008/05/19	Neg

23	23	Alt Ruppin	2008/05/19	Neg
24	24	Alt Ruppin	2008/05/19	Neg
25	25	Alt Ruppin	2008/05/19	Neg
26	26	Alt Ruppin	2008/05/19	Neg
27	27	Alt Ruppin	2008/05/19	Neg
28	28	Alt Ruppin	2008/05/19	Neg
29	33	Alt Ruppin	2008/05/19	Neg
30	34	Alt Ruppin	2008/05/19	Neg
31	35	Alt Ruppin	2008/05/19	Neg
32	36	Alt Ruppin	2008/05/19	Neg
33	1	Doberlug-Kirchhain	2008/06/13	Neg
34	2	Doberlug-Kirchhain	2008/06/13	Neg
35	3	Doberlug-Kirchhain	2008/06/13	Neg
36	4	Doberlug-Kirchhain	2008/06/13	Neg
37	5	Doberlug-Kirchhain	2008/06/13	Neg
38	6	Doberlug-Kirchhain	2008/06/13	Neg
39	7	Doberlug-Kirchhain	2008/06/13	Neg
40	8	Doberlug-Kirchhain	2008/06/13	Neg
41	9	Doberlug-Kirchhain	2008/06/13	Neg
42	10	Doberlug-Kirchhain	2008/06/13	Neg
43	11	Doberlug-Kirchhain	2008/06/13	Neg
44	12	Doberlug-Kirchhain	2008/06/13	Neg
45	13	Doberlug-Kirchhain	2008/06/13	Neg
46	14	Doberlug-Kirchhain	2008/06/13	Neg
47	15	Doberlug-Kirchhain	2008/06/13	Neg
48	16	Doberlug-Kirchhain	2008/06/13	Neg
49	17	Doberlug-Kirchhain	2008/06/13	Neg
50	18	Doberlug-Kirchhain	2008/06/13	Neg
51	19	Doberlug-Kirchhain	2008/06/13	Neg
52	20	Doberlug-Kirchhain	2008/06/13	Neg
53	21	Doberlug-Kirchhain	2008/06/13	Neg
54	22	Doberlug-Kirchhain	2008/06/13	Neg
55	23	Doberlug-Kirchhain	2008/06/13	Neg
56	24	Doberlug-Kirchhain	2008/06/13	Neg
57	25	Doberlug-Kirchhain	2008/06/13	Neg
58	26	Doberlug-Kirchhain	2008/06/13	Neg
59	27	Doberlug-Kirchhain	2008/06/13	Neg
60	28	Doberlug-Kirchhain	2008/06/13	Neg
61	29	Doberlug-Kirchhain	2008/06/13	Neg
62	30	Doberlug-Kirchhain	2008/06/13	Neg
63	31	Doberlug-Kirchhain	2008/06/13	Neg
64	32	Doberlug-Kirchhain	2008/06/13	Neg
65	33	Doberlug-Kirchhain	2008/06/13	Neg
66	34	Doberlug-Kirchhain	2008/06/13	Neg
67	35	Doberlug-Kirchhain	2008/06/13	Neg
68	36	Doberlug-Kirchhain	2008/06/13	Neg
69	1	Belzig	2008/06/16	Neg
70	2	Belzig	2008/06/16	Neg
71	3	Belzig	2008/06/16	Neg
72	4	Belzig	2008/06/16	Neg
73	5	Belzig	2008/06/16	Neg
74	6	Belzig	2008/06/16	Neg
75	7	Belzig	2008/06/16	Neg
76	8	Belzig	2008/06/16	Neg
77	9	Belzig	2008/06/16	Neg
78	10	Belzig	2008/06/16	Neg
79	11	Belzig	2008/06/16	Neg
80	12	Belzig	2008/06/16	Neg
81	13	Belzig	2008/06/16	Neg
82	14	Belzig	2008/06/16	Neg
83	15	Belzig	2008/06/16	Neg
84	16	Belzig	2008/06/16	Neg
85	17	Belzig	2008/06/16	Pos

86	18	Belzig	2008/06/16	Neg
87	19	Belzig	2008/06/16	Neg
88	20	Belzig	2008/06/16	Neg
89	21	Belzig	2008/06/16	Neg
90	22	Belzig	2008/06/16	Neg
91	23	Belzig	2008/06/16	Neg
92	24	Belzig	2008/06/16	Neg
93	25	Belzig	2008/06/16	Neg
94	26	Belzig	2008/06/16	Neg
95	27	Belzig	2008/06/16	Neg
96	28	Belzig	2008/06/16	Neg
97	29	Belzig	2008/06/16	Neg
98	30	Belzig	2008/06/16	Neg
99	31	Belzig	2008/06/16	Neg
100	32	Belzig	2008/06/16	Neg
101	33	Belzig	2008/06/16	Neg
102	34	Belzig	2008/06/16	Neg
103	35	Belzig	2008/06/16	Neg
104	36	Belzig	2008/06/16	Neg
105	37	Belzig	2008/06/16	Neg
106	38	Belzig	2008/06/16	Neg
107	39	Belzig	2008/06/16	Neg
108	40	Belzig	2008/06/16	Neg
109	41	Belzig	2008/06/16	Neg
110	42	Belzig	2008/06/16	Neg
111	43	Belzig	2008/06/16	Neg
112	44	Belzig	2008/06/16	Neg
113	45	Belzig	2008/06/16	Neg
114	46	Belzig	2008/06/16	Neg
115	47	Belzig	2008/06/16	Neg
116	48	Belzig	2008/06/16	Neg
117	49	Belzig	2008/06/16	Neg
118	50	Belzig	2008/06/16	Neg
119	51	Belzig	2008/06/16	Neg
120	52	Belzig	2008/06/16	Neg
121	53	Belzig	2008/06/16	Neg
122	54	Belzig	2008/06/16	Neg
123	55	Belzig	2008/06/16	Neg
124	56	Belzig	2008/06/16	Neg
125	57	Belzig	2008/06/16	Neg
126	58	Belzig	2008/06/16	Neg
127	59	Belzig	2008/06/16	Neg
128	60	Belzig	2008/06/16	Neg
129	61	Belzig	2008/06/16	Neg
130	62	Belzig	2008/06/16	Neg
131	63	Belzig	2008/06/16	Neg
132	64	Belzig	2008/06/16	Neg
133	65	Belzig	2008/06/16	Neg
134	66	Belzig	2008/06/16	Neg
135	67	Belzig	2008/06/16	Neg
136	68	Belzig	2008/06/16	Neg
137	69	Belzig	2008/06/16	Neg
138	70	Belzig	2008/06/16	Neg
139	71	Belzig	2008/06/16	Neg
140	1	Wünsdorf	2008/06/20	Neg
141	2	Wünsdorf	2008/06/20	Neg
142	3	Wünsdorf	2008/06/20	Neg
143	4	Wünsdorf	2008/06/20	Neg
144	5	Wünsdorf	2008/06/20	Neg
145	6	Wünsdorf	2008/06/20	Neg
146	7	Wünsdorf	2008/06/20	Neg
147	8	Wünsdorf	2008/06/20	Neg
148	9	Wünsdorf	2008/06/20	Pos

149	10	Wünsdorf	2008/06/20	Neg
150	11	Wünsdorf	2008/06/20	Neg
151	12	Wünsdorf	2008/06/20	Neg
152	13	Wünsdorf	2008/06/20	Neg
153	14	Wünsdorf	2008/06/20	Neg
154	15	Wünsdorf	2008/06/20	Neg
155	16	Wünsdorf	2008/06/20	Neg
156	17	Wünsdorf	2008/06/20	Neg
157	18	Wünsdorf	2008/06/20	Neg
158	19	Wünsdorf	2008/06/20	Neg
159	20	Wünsdorf	2008/06/20	Neg
160	21	Wünsdorf	2008/06/20	Neg
161	22	Wünsdorf	2008/06/20	Neg
162	23	Wünsdorf	2008/06/20	Neg
163	24	Wünsdorf	2008/06/20	Neg
164	25	Wünsdorf	2008/06/20	Neg
165	26	Wünsdorf	2008/06/20	Neg
166	27	Wünsdorf	2008/06/20	Neg
167	28	Wünsdorf	2008/06/20	Neg
168	29	Wünsdorf	2008/06/20	Neg
169	30	Wünsdorf	2008/06/20	Neg
170	31	Wünsdorf	2008/06/20	Neg
171	32	Wünsdorf	2008/06/20	Neg
172	33	Wünsdorf	2008/06/20	Neg
173	34	Wünsdorf	2008/06/20	Neg
174	35	Wünsdorf	2008/06/20	Neg
175	36	Wünsdorf	2008/06/20	Neg
176	37	Wünsdorf	2008/06/20	Neg
177	38	Wünsdorf	2008/06/20	Neg
178	39	Wünsdorf	2008/06/20	Neg
179	40	Wünsdorf	2008/06/20	Neg
180	41	Wünsdorf	2008/06/20	Neg
181	42	Wünsdorf	2008/06/20	Neg
182	43	Wünsdorf	2008/06/20	Neg
183	44	Wünsdorf	2008/06/20	Neg
184	45	Wünsdorf	2008/06/20	Neg
185	46	Wünsdorf	2008/06/20	Neg
186	47	Wünsdorf	2008/06/20	Neg
187	48	Wünsdorf	2008/06/20	Neg
188	49	Wünsdorf	2008/06/20	Neg
189	50	Wünsdorf	2008/06/20	Neg
190	51	Wünsdorf	2008/06/20	Neg
191	52	Wünsdorf	2008/06/20	Neg
192	53	Wünsdorf	2008/06/20	Neg
193	54	Wünsdorf	2008/06/20	Neg
194	55	Wünsdorf	2008/06/20	Neg
195	56	Wünsdorf	2008/06/20	Neg
196	57	Wünsdorf	2008/06/20	Neg
197	58	Wünsdorf	2008/06/20	Neg
198	59	Wünsdorf	2008/06/20	Neg
199	60	Wünsdorf	2008/06/20	Neg
200	61	Wünsdorf	2008/06/20	Neg
201	62	Wünsdorf	2008/06/20	Neg
202	63	Wünsdorf	2008/06/20	Neg
203	64	Wünsdorf	2008/06/20	Neg
204	65	Wünsdorf	2008/06/20	Neg
205	66	Wünsdorf	2008/06/20	Neg
206	67	Wünsdorf	2008/06/20	Neg
207	68	Wünsdorf	2008/06/20	Neg
208	69	Wünsdorf	2008/06/20	Neg
209	70	Wünsdorf	2008/06/20	Neg
210	71	Wünsdorf	2008/06/20	Neg
211	72	Wünsdorf	2008/06/20	Neg

212	73	Wünsdorf	2008/06/20	Neg
213	74	Wünsdorf	2008/06/20	Neg
214	75	Wünsdorf	2008/06/20	Neg
215	76	Wünsdorf	2008/06/20	Neg
216	77	Wünsdorf	2008/06/20	Neg
217	78	Wünsdorf	2008/06/20	Neg
218	79	Wünsdorf	2008/06/20	Neg
219	80	Wünsdorf	2008/06/20	Neg
220	81	Wünsdorf	2008/06/20	Neg
221	82	Wünsdorf	2008/06/20	Neg
222	83	Wünsdorf	2008/06/20	Neg
223	84	Wünsdorf	2008/06/20	Neg
224	85	Wünsdorf	2008/06/20	Neg
225	86	Wünsdorf	2008/06/20	Neg
226	87	Wünsdorf	2008/06/20	Neg
227	88	Wünsdorf	2008/06/20	Neg
228	89	Wünsdorf	2008/06/20	Neg
229	90	Wünsdorf	2008/06/20	Neg
230	91	Wünsdorf	2008/06/20	Neg
231	92	Wünsdorf	2008/06/20	Neg
232	93	Wünsdorf	2008/06/20	Neg
233	94	Wünsdorf	2008/06/20	Neg
234	95	Wünsdorf	2008/06/20	Neg
235	96	Wünsdorf	2008/06/20	Neg
236	97	Wünsdorf	2008/06/20	Neg
237	98	Wünsdorf	2008/06/20	Neg
238	99	Wünsdorf	2008/06/20	Neg
239	100	Wünsdorf	2008/06/20	Neg
240	101	Wünsdorf	2008/06/20	Neg
241	1	Lübben	2008/06/23	Neg
242	2	Lübben	2008/06/23	Neg
243	3	Lübben	2008/06/23	Neg
244	4	Lübben	2008/06/23	Neg
245	5	Lübben	2008/06/23	Neg
246	6	Lübben	2008/06/23	Neg
247	7	Lübben	2008/06/23	Neg
248	8	Lübben	2008/06/23	Neg
249	9	Lübben	2008/06/23	Neg
250	10	Lübben	2008/06/23	Neg
251	11	Lübben	2008/06/23	Neg
252	12	Lübben	2008/06/23	Neg
253	13	Lübben	2008/06/23	Neg
254	14	Lübben	2008/06/23	Neg
255	15	Lübben	2008/06/23	Neg
256	16	Lübben	2008/06/23	Neg
257	17	Lübben	2008/06/23	Neg
258	18	Lübben	2008/06/23	Neg
259	19	Lübben	2008/06/23	Neg
260	20	Lübben	2008/06/23	Neg
261	21	Lübben	2008/06/23	Neg
262	22	Lübben	2008/06/23	Neg
263	23	Lübben	2008/06/23	Neg
264	24	Lübben	2008/06/23	Neg
265	25	Lübben	2008/06/23	Neg
266	26	Lübben	2008/06/23	Neg
267	27	Lübben	2008/06/23	Neg
268	28	Lübben	2008/06/23	Neg
269	29	Lübben	2008/06/23	Neg
270	30	Lübben	2008/06/23	Neg
271	31	Lübben	2008/06/23	Neg
272	32	Lübben	2008/06/23	Neg
273	33	Lübben	2008/06/23	Neg
274	34	Lübben	2008/06/23	Neg

275	35	Lübben	2008/06/23	Neg
276	36	Lübben	2008/06/23	Neg
277	37	Lübben	2008/06/23	Neg
278	38	Lübben	2008/06/23	Neg
279	39	Lübben	2008/06/23	Neg
280	40	Lübben	2008/06/23	Neg
281	41	Lübben	2008/06/23	Neg
282	42	Lübben	2008/06/23	Pos
283	43	Lübben	2008/06/23	Neg
284	44	Lübben	2008/06/23	Neg
285	45	Lübben	2008/06/23	Neg
286	46	Lübben	2008/06/23	Neg
287	1	Kyritz	2008/06/24	Neg
288	2	Kyritz	2008/06/24	Neg
289	3	Kyritz	2008/06/24	Neg
290	4	Kyritz	2008/06/24	Neg
291	5	Kyritz	2008/06/24	Neg
292	6	Kyritz	2008/06/24	Neg
293	7	Kyritz	2008/06/24	Neg
294	8	Kyritz	2008/06/24	Neg
295	9	Kyritz	2008/06/24	Neg
296	10	Kyritz	2008/06/24	Neg
297	11	Kyritz	2008/06/24	Neg
298	12	Kyritz	2008/06/24	Neg
299	13	Kyritz	2008/06/24	Neg
300	14	Kyritz	2008/06/24	Neg
301	15	Kyritz	2008/06/24	Neg
302	16	Kyritz	2008/06/24	Neg
303	17	Kyritz	2008/06/24	Neg
304	18	Kyritz	2008/06/24	Neg
305	19	Kyritz	2008/06/24	Neg
306	20	Kyritz	2008/06/24	Neg
307	21	Kyritz	2008/06/24	Neg
308	22	Kyritz	2008/06/24	Neg
309	23	Kyritz	2008/06/24	Neg
310	24	Kyritz	2008/06/24	Neg
311	25	Kyritz	2008/06/24	Neg
312	26	Kyritz	2008/06/24	Neg
313	27	Kyritz	2008/06/24	Neg
314	28	Kyritz	2008/06/24	Neg
315	29	Kyritz	2008/06/24	Neg
316	30	Kyritz	2008/06/24	Neg
317	31	Kyritz	2008/06/24	Neg
318	32	Kyritz	2008/06/24	Neg
319	33	Kyritz	2008/06/24	Neg
320	34	Kyritz	2008/06/24	Neg
321	35	Kyritz	2008/06/24	Neg
322	36	Kyritz	2008/06/24	Neg
323	37	Kyritz	2008/06/24	Neg
324	38	Kyritz	2008/06/24	Neg
325	39	Kyritz	2008/06/24	Neg
326	40	Kyritz	2008/06/24	Neg
327	41	Kyritz	2008/06/24	Neg
328	42	Kyritz	2008/06/24	Neg
329	43	Kyritz	2008/06/24	Neg
330	44	Kyritz	2008/06/24	Neg
331	45	Kyritz	2008/06/24	Neg
332	46	Kyritz	2008/06/24	Neg
333	47	Kyritz	2008/06/24	Neg
334	1	Peitz	2008/06/24	Neg
335	2	Peitz	2008/06/24	Neg
336	3	Peitz	2008/06/24	Neg
337	4	Peitz	2008/06/24	Neg

338	5	Peitz	2008/06/24	Neg
339	6	Peitz	2008/06/24	Neg
340	7	Peitz	2008/06/24	Neg
341	8	Peitz	2008/06/24	Neg
342	9	Peitz	2008/06/24	Neg
343	10	Peitz	2008/06/24	Neg
344	11	Peitz	2008/06/24	Neg
345	12	Peitz	2008/06/24	Neg
346	13	Peitz	2008/06/24	Neg
347	14	Peitz	2008/06/24	Neg
348	15	Peitz	2008/06/24	Neg
349	16	Peitz	2008/06/24	Neg
350	17	Peitz	2008/06/24	Neg
351	18	Peitz	2008/06/24	Neg
352	19	Peitz	2008/06/24	Neg
353	20	Peitz	2008/06/24	Neg
354	21	Peitz	2008/06/24	Neg
355	22	Peitz	2008/06/24	Neg
356	23	Peitz	2008/06/24	Neg
357	24	Peitz	2008/06/24	Neg
358	25	Peitz	2008/06/24	Neg
359	26	Peitz	2008/06/24	Neg
360	27	Peitz	2008/06/24	Neg
361	28	Peitz	2008/06/24	Neg
362	29	Peitz	2008/06/24	Neg
363	30	Peitz	2008/06/24	Neg
364	31	Peitz	2008/06/24	Neg
365	32	Peitz	2008/06/24	Neg
366	33	Peitz	2008/06/24	Neg
367	34	Peitz	2008/06/24	Neg
368	35	Peitz	2008/06/24	Neg
369	36	Peitz	2008/06/24	Neg
370	37	Peitz	2008/06/24	Neg
371	38	Peitz	2008/06/24	Neg
372	39	Peitz	2008/06/24	Neg
373	40	Peitz	2008/06/24	Neg
374	41	Peitz	2008/06/24	Neg
375	42	Peitz	2008/06/24	Neg
376	43	Peitz	2008/06/24	Neg
377	44	Peitz	2008/06/24	Neg
378	45	Peitz	2008/06/24	Neg
379	46	Peitz	2008/06/24	Neg
380	47	Peitz	2008/06/24	Neg
381	48	Peitz	2008/06/24	Neg
382	49	Peitz	2008/06/24	Neg
383	50	Peitz	2008/06/24	Neg
384	51	Peitz	2008/06/24	Neg
385	52	Peitz	2008/06/24	Neg
386	53	Peitz	2008/06/24	Neg
387	54	Peitz	2008/06/24	Neg
388	55	Peitz	2008/06/24	Neg
389	56	Peitz	2008/06/24	Neg
390	1	Eberswalde	2008/06/25	Neg
391	2	Eberswalde	2008/06/25	Neg
392	3	Eberswalde	2008/06/25	Neg
393	4	Eberswalde	2008/06/25	Pos
394	5	Eberswalde	2008/06/25	Neg
395	6	Eberswalde	2008/06/25	Neg
396	7	Eberswalde	2008/06/25	Neg
397	8	Eberswalde	2008/06/25	Neg
398	9	Eberswalde	2008/06/25	Neg
399	10	Eberswalde	2008/06/25	Neg
400	11	Eberswalde	2008/06/25	Neg

401	12	Eberswalde	2008/06/25	Neg
402	13	Eberswalde	2008/06/25	Neg
403	14	Eberswalde	2008/06/25	Neg
404	15	Eberswalde	2008/06/25	Neg
405	16	Eberswalde	2008/06/25	Neg
406	17	Eberswalde	2008/06/25	Pos
407	18	Eberswalde	2008/06/25	Neg
408	19	Eberswalde	2008/06/25	Neg
409	20	Eberswalde	2008/06/25	Neg
410	21	Eberswalde	2008/06/25	Neg
411	22	Eberswalde	2008/06/25	Neg
412	23	Eberswalde	2008/06/25	Neg
413	24	Eberswalde	2008/06/25	Neg
414	25	Eberswalde	2008/06/25	Neg
415	26	Eberswalde	2008/06/25	Neg
416	27	Eberswalde	2008/06/25	Neg
417	28	Eberswalde	2008/06/25	Neg
418	29	Eberswalde	2008/06/25	Neg
419	30	Eberswalde	2008/06/25	Neg
420	31	Eberswalde	2008/06/25	Neg
421	32	Eberswalde	2008/06/25	Neg
422	33	Eberswalde	2008/06/25	Neg
423	34	Eberswalde	2008/06/25	Neg
424	35	Eberswalde	2008/06/25	Neg
425	36	Eberswalde	2008/06/25	Neg
426	37	Eberswalde	2008/06/25	Neg
427	38	Eberswalde	2008/06/25	Neg
428	39	Eberswalde	2008/06/25	Neg
429	40	Eberswalde	2008/06/25	Neg
430	41	Eberswalde	2008/06/25	Neg
431	42	Eberswalde	2008/06/25	Neg
432	43	Eberswalde	2008/06/25	Neg
433	44	Eberswalde	2008/06/25	Neg
434	45	Eberswalde	2008/06/25	Neg
435	46	Eberswalde	2008/06/25	Neg
436	47	Eberswalde	2008/06/25	Neg
437	48	Eberswalde	2008/06/25	Neg
438	49	Eberswalde	2008/06/25	Neg
439	50	Eberswalde	2008/06/25	Neg
440	51	Eberswalde	2008/06/25	Neg
441	52	Eberswalde	2008/06/25	Neg
442	53	Eberswalde	2008/06/25	Neg
443	54	Eberswalde	2008/06/25	Neg
444	55	Eberswalde	2008/06/25	Neg
445	56	Eberswalde	2008/06/25	Neg
446	57	Eberswalde	2008/06/25	Neg
447	58	Eberswalde	2008/06/25	Neg
448	59	Eberswalde	2008/06/25	Neg
449	60	Eberswalde	2008/06/25	Neg
450	61	Eberswalde	2008/06/25	Neg
451	62	Eberswalde	2008/06/25	Neg
452	63	Eberswalde	2008/06/25	Neg
453	64	Eberswalde	2008/06/25	Neg
454	65	Eberswalde	2008/06/25	Neg
455	66	Eberswalde	2008/06/25	Neg
456	67	Eberswalde	2008/06/25	Neg
457	68	Eberswalde	2008/06/25	Neg
458	69	Eberswalde	2008/06/25	Neg
459	70	Eberswalde	2008/06/25	Neg
460	71	Eberswalde	2008/06/25	Neg
461	72	Eberswalde	2008/06/25	Neg
462	73	Eberswalde	2008/06/25	Neg
463	74	Eberswalde	2008/06/25	Neg

464	75	Eberswalde	2008/06/25	Neg
465	76	Eberswalde	2008/06/25	Neg
466	77	Eberswalde	2008/06/25	Neg
467	1	Templin	2008/06/26	Neg
468	2	Templin	2008/06/26	Neg
469	3	Templin	2008/06/26	Neg
470	4	Templin	2008/06/26	Neg
471	5	Templin	2008/06/26	Neg
472	6	Templin	2008/06/26	Neg
473	7	Templin	2008/06/26	Neg
474	8	Templin	2008/06/26	Neg
475	9	Templin	2008/06/26	Pos
476	10	Templin	2008/06/26	Neg
477	11	Templin	2008/06/26	Neg
478	12	Templin	2008/06/26	Neg
479	13	Templin	2008/06/26	Neg
480	14	Templin	2008/06/26	Neg
481	15	Templin	2008/06/26	Neg
482	16	Templin	2008/06/26	Neg
483	17	Templin	2008/06/26	Neg
484	18	Templin	2008/06/26	Neg
485	19	Templin	2008/06/26	Neg
486	20	Templin	2008/06/26	Neg
487	21	Templin	2008/06/26	Neg
488	22	Templin	2008/06/26	Neg
489	23	Templin	2008/06/26	Neg
490	24	Templin	2008/06/26	Neg
491	25	Templin	2008/06/26	Neg
492	26	Templin	2008/06/26	Neg
493	27	Templin	2008/06/26	Neg
494	28	Templin	2008/06/26	Neg
495	29	Templin	2008/06/26	Neg
496	30	Templin	2008/06/26	Neg
497	31	Templin	2008/06/26	Neg
498	32	Templin	2008/06/26	Neg
499	33	Templin	2008/06/26	Neg
500	34	Templin	2008/06/26	Neg
501	35	Templin	2008/06/26	Neg
502	36	Templin	2008/06/26	Neg
503	37	Templin	2008/06/26	Neg
504	38	Templin	2008/06/26	Neg
505	39	Templin	2008/06/26	Neg
506	40	Templin	2008/06/26	Neg
507	41	Templin	2008/06/26	Neg
508	42	Templin	2008/06/26	Neg
509	43	Templin	2008/06/26	Neg
510	44	Templin	2008/06/26	Neg
511	45	Templin	2008/06/26	Neg
512	46	Templin	2008/06/26	Neg
513	47	Templin	2008/06/26	Neg
514	48	Templin	2008/06/26	Neg
515	49	Templin	2008/06/26	Neg
516	50	Templin	2008/06/26	Neg
517	51	Templin	2008/06/26	Neg
518	52	Templin	2008/06/26	Neg
519	1	Müllrose	2008/06/30	Neg
520	2	Müllrose	2008/06/30	Neg
521	3	Müllrose	2008/06/30	Neg
522	4	Müllrose	2008/06/30	Neg
523	5	Müllrose	2008/06/30	Neg
524	6	Müllrose	2008/06/30	Neg
525	7	Müllrose	2008/06/30	Neg
526	8	Müllrose	2008/06/30	Neg

527	9	Müllrose	2008/06/30	Neg
528	10	Müllrose	2008/06/30	Neg
529	11	Müllrose	2008/06/30	Neg
530	12	Müllrose	2008/06/30	Neg
531	13	Müllrose	2008/06/30	Neg
532	14	Müllrose	2008/06/30	Neg
533	15	Müllrose	2008/06/30	Neg
534	16	Müllrose	2008/06/30	Neg
535	17	Müllrose	2008/06/30	Neg
536	18	Müllrose	2008/06/30	Neg
537	19	Müllrose	2008/06/30	Neg
538	20	Müllrose	2008/06/30	Neg
539	21	Müllrose	2008/06/30	Neg
540	22	Müllrose	2008/06/30	Neg
541	23	Müllrose	2008/06/30	Neg
542	24	Müllrose	2008/06/30	Neg
543	25	Müllrose	2008/06/30	Neg
544	26	Müllrose	2008/06/30	Neg
545	27	Müllrose	2008/06/30	Neg
546	28	Müllrose	2008/06/30	Neg
547	29	Müllrose	2008/06/30	Neg
548	30	Müllrose	2008/06/30	Neg
549	31	Müllrose	2008/06/30	Neg
550	32	Müllrose	2008/06/30	Neg
551	33	Müllrose	2008/06/30	Neg
552	34	Müllrose	2008/06/30	Neg
553	35	Müllrose	2008/06/30	Neg
554	36	Müllrose	2008/06/30	Neg
555	37	Müllrose	2008/06/30	Neg
556	38	Müllrose	2008/06/30	Neg
557	39	Müllrose	2008/06/30	Neg
558	40	Müllrose	2008/06/30	Neg
559	41	Müllrose	2008/06/30	Neg
560	42	Müllrose	2008/06/30	Neg
561	43	Müllrose	2008/06/30	Neg
562	44	Müllrose	2008/06/30	Neg
563	45	Müllrose	2008/06/30	Neg

Neg, negative; Pos, positive

Table S2: Detection of LCMV-reactive antibodies in house mice from different regions in Germany.

KS_Number	Federal state	Site	Area Code	Family	Genus	Species	Capture Date	LCMV	Dilution	Trapping published in
Mu09/0244	Baden-Wuerttemberg	Stuttgart	70376	Muridae	*Mus*	*musculus*	2008/09/10	Neg		Gertler et al. 2017; Pelz et al. 2010
Mu09/0245	Baden-Wuerttemberg	Stuttgart	70376	Muridae	*Mus*	*musculus*	2008/09/10	Pos	1:4	Gertler et al. 2017; Pelz et al. 2010
Mu09/0246	Baden-Wuerttemberg	Stuttgart	70376	Muridae	*Mus*	*musculus*		Neg		Gertler et al. 2017; Pelz et al. 2010
Mu09/0247	Baden-Wuerttemberg	Stuttgart	70376	Muridae	*Mus*	*musculus*		Neg		Gertler et al. 2017; Pelz et al. 2010
Mu09/0248	Baden-Wuerttemberg	Stuttgart	70376	Muridae	*Mus*	*musculus*	2008/09/27	Neg		Gertler et al. 2017; Pelz et al. 2010
Mu09/0249	Baden-Wuerttemberg	Stuttgart	70376	Muridae	*Mus*	*musculus*	2008/09/27	Neg		Gertler et al. 2017; Pelz et al. 2010
Mu09/0250	Baden-Wuerttemberg	Stuttgart	70376	Muridae	*Mus*	*musculus*	2008/09/27	Neg		Gertler et al. 2017; Pelz et al. 2010
Mu09/0252	Baden-Wuerttemberg	Stuttgart	70376	Muridae	*Mus*	*musculus*	2008/09/28	Neg		Gertler et al. 2017; Pelz et al. 2010
Mu09/0254	Baden-Wuerttemberg	Stuttgart	70376	Muridae	*Mus*	*musculus*	2008/09/28	Neg		Gertler et al. 2017; Pelz et al. 2010
Mu09/0255	Baden-Wuerttemberg	Stuttgart	70376	Muridae	*Mus*	*musculus*		Neg		Gertler et al. 2017; Pelz et al. 2010
Mu09/0256	Baden-Wuerttemberg	Stuttgart	70376	Muridae	*Mus*	*musculus*	2008/09/25	Neg		Gertler et al. 2017; Pelz et al. 2010
Mu09/0262	Baden-Wuerttemberg	Stuttgart	70376	Muridae	*Mus*	*musculus*	2008/09/25	Neg		Gertler et al. 2017; Pelz et al. 2010
Mu09/0263	Baden-Wuerttemberg	Stuttgart	70376	Muridae	*Mus*	*musculus*	2008/07/06	Neg		Gertler et al. 2017; Pelz et al. 2010
Mu09/0267	Baden-Wuerttemberg	Stuttgart	70376	Muridae	*Mus*	*musculus*	2008/10/02	Neg		Gertler et al. 2017; Pelz et al. 2010
Mu09/0268	Baden-Wuerttemberg	Stuttgart	70376	Muridae	*Mus*	*musculus*	2008/10/02	Neg		Gertler et al. 2017; Pelz et al. 2010
Mu09/0269	Baden-Wuerttemberg	Stuttgart	70376	Muridae	*Mus*	*musculus*	2008/10/02	Neg		Gertler et al. 2017; Pelz et al. 2010
Mu09/0270	Baden-Wuerttemberg	Stuttgart	70376	Muridae	*Mus*	*musculus*	2008/10/02	Neg		Gertler et al. 2017; Pelz et al. 2010
Mu09/0271	Baden-Wuerttemberg	Stuttgart	70376	Muridae	*Mus*	*musculus*		Neg		Gertler et al. 2017; Pelz et al. 2010
Mu09/0272	Baden-Wuerttemberg	Stuttgart	70376	Muridae	*Mus*	*musculus*		Neg		Gertler et al. 2017; Pelz et al. 2010
Mu09/0283	Baden-Wuerttemberg	Stuttgart	70173	Muridae	*Mus*	*musculus*	2008/10/01	Neg		Gertler et al. 2017; Pelz et al. 2010
Mu09/0284	Baden-Wuerttemberg	Stuttgart	70173	Muridae	*Mus*	*musculus*	2008/10/01	Neg		Gertler et al. 2017; Pelz et al. 2010
Mu09/0285	Baden-Wuerttemberg	Stuttgart	70173	Muridae	*Mus*	*musculus*	2008/10/01	Neg		Gertler et al. 2017; Pelz et al. 2010
Mu09/0286	Baden-Wuerttemberg	Stuttgart	70173	Muridae	*Mus*	*musculus*	2008/09/22	Neg		Gertler et al. 2017; Pelz et al. 2010
Mu09/0287	Baden-Wuerttemberg	Stuttgart	70173	Muridae	*Mus*	*musculus*	2008/09/29	Neg		Gertler et al. 2017; Pelz et al. 2010
Mu09/0288	Baden-Wuerttemberg	Stuttgart	70173	Muridae	*Mus*	*musculus*	2008/09/29	Neg		Gertler et al. 2017; Pelz et al. 2010
Mu09/0289	Baden-Wuerttemberg	Stuttgart	70173	Muridae	*Mus*	*musculus*	2008/09/29	Neg		Gertler et al. 2017; Pelz et al. 2010
Mu09/0301	Baden-Wuerttemberg	Stuttgart	70173	Muridae	*Mus*	*musculus*	2008/10/14	Neg		Gertler et al. 2017; Pelz et al. 2010

Mu09/0302	Baden-Wuerttemberg	Stuttgart	70173	Muridae	*Mus*	*musculus*	2008/10/14	Neg		Gertler et al. 2017; Pelz et al. 2010
Mu09/0306	Baden-Wuerttemberg	Stuttgart	70173	Muridae	*Mus*	*musculus*	2008/10/17	Neg		Gertler et al. 2017; Pelz et al. 2010
Mu09/0307	Baden-Wuerttemberg	Stuttgart	70173	Muridae	*Mus*	*musculus*	2008/10/17	Neg		Gertler et al. 2017; Pelz et al. 2010
Mu09/0313	Baden-Wuerttemberg	Stuttgart	70173	Muridae	*Mus*	*musculus*	2008/10/21	Neg		Gertler et al. 2017; Pelz et al. 2010
Mu09/0314	Baden-Wuerttemberg	Stuttgart	70173	Muridae	*Mus*	*musculus*	2008/10/21	Neg		Gertler et al. 2017; Pelz et al. 2010
Mu09/0315	Baden-Wuerttemberg	Stuttgart	70173	Muridae	*Mus*	*musculus*	2008/10/23	Neg		Gertler et al. 2017; Pelz et al. 2010
Mu09/0316	Baden-Wuerttemberg	Stuttgart	70173	Muridae	*Mus*	*musculus*	2008/10/23	Neg		Gertler et al. 2017; Pelz et al. 2010
Mu09/0317	Baden-Wuerttemberg	Stuttgart	70173	Muridae	*Mus*	*musculus*	2008/10/24	Neg		Gertler et al. 2017; Pelz et al. 2010
Mu09/0318	Baden-Wuerttemberg	Stuttgart	70173	Muridae	*Mus*	*musculus*	2008/10/24	Neg		Gertler et al. 2017; Pelz et al. 2010
Mu09/0319	Baden-Wuerttemberg	Stuttgart	70173	Muridae	*Mus*	*musculus*	2008/10/24	Neg		Gertler et al. 2017; Pelz et al. 2010
Mu09/0325	Baden-Wuerttemberg	Stuttgart	70173	Muridae	*Mus*	*musculus*	2008/10/27	Neg		Gertler et al. 2017; Pelz et al. 2010
Mu09/0326	Baden-Wuerttemberg	Stuttgart	70173	Muridae	*Mus*	*musculus*	2008/10/27	Neg		Gertler et al. 2017; Pelz et al. 2010
Mu09/0327	Baden-Wuerttemberg	Stuttgart	70173	Muridae	*Mus*	*musculus*	2008/10/28	Neg		Gertler et al. 2017; Pelz et al. 2010
Mu09/0328	Baden-Wuerttemberg	Stuttgart	70173	Muridae	*Mus*	*musculus*	2008/10/28	Neg		Gertler et al. 2017; Pelz et al. 2010
Mu09/0329	Baden-Wuerttemberg	Stuttgart	70173	Muridae	*Mus*	*musculus*	2008/10/28	Neg		Gertler et al. 2017; Pelz et al. 2010
Mu09/0330	Baden-Wuerttemberg	Stuttgart	70173	Muridae	*Mus*	*musculus*	2008/10/28	Neg		Gertler et al. 2017; Pelz et al. 2010
Mu09/0331	Baden-Wuerttemberg	Stuttgart	70173	Muridae	*Mus*	*musculus*	2008/10/28	Neg		Gertler et al. 2017; Pelz et al. 2010
Mu09/0332	Baden-Wuerttemberg	Stuttgart	70173	Muridae	*Mus*	*musculus*	2008/10/28	Neg		Gertler et al. 2017; Pelz et al. 2010
Mu09/0333	Baden-Wuerttemberg	Stuttgart	70173	Muridae	*Mus*	*musculus*	2008/10/30	Neg		Gertler et al. 2017; Pelz et al. 2010
Mu09/0335	Baden-Wuerttemberg	Stuttgart	70173	Muridae	*Mus*	*musculus*	2008/10/31	Neg		Gertler et al. 2017; Pelz et al. 2010
Mu09/0336	Baden-Wuerttemberg	Stuttgart	70173	Muridae	*Mus*	*musculus*	2008/10/30	Neg		Gertler et al. 2017; Pelz et al. 2010
Mu09/0337	Baden-Wuerttemberg	Stuttgart	70173	Muridae	*Mus*	*musculus*	2008/10/30	Neg		Gertler et al. 2017; Pelz et al. 2010
Mu09/0338	Baden-Wuerttemberg	Stuttgart	70173	Muridae	*Mus*	*musculus*	2008/10/30	Neg		Gertler et al. 2017; Pelz et al. 2010
Mu09/0339	Baden-Wuerttemberg	Stuttgart	70173	Muridae	*Mus*	*musculus*	2008/10/30	Neg		Gertler et al. 2017; Pelz et al. 2010
Mu09/0340	Baden-Wuerttemberg	Stuttgart	70173	Muridae	*Mus*	*musculus*	2008/10/30	Neg		Gertler et al. 2017; Pelz et al. 2010
Mu09/0341	Baden-Wuerttemberg	Stuttgart	70173	Muridae	*Mus*	*musculus*	2008/10/30	Neg		Gertler et al. 2017; Pelz et al. 2010
Mu09/0342	Baden-Wuerttemberg	Stuttgart	70173	Muridae	*Mus*	*musculus*	2008/10/30	Neg		Gertler et al. 2017; Pelz et al. 2010
Mu09/0343	Baden-Wuerttemberg	Stuttgart	70173	Muridae	*Mus*	*musculus*	2008/10/30	Neg		Gertler et al. 2017; Pelz et al. 2010
Mu09/0344	Baden-Wuerttemberg	Stuttgart	70173	Muridae	*Mus*	*musculus*	2008/11/01	Neg		Gertler et al. 2017; Pelz et al. 2010
Mu09/0352	Baden-Wuerttemberg	Stuttgart	70173	Muridae	*Mus*	*musculus*	2008/11/03	Neg		Gertler et al. 2017; Pelz et al. 2010
Mu09/0353	Baden-Wuerttemberg	Stuttgart	70173	Muridae	*Mus*	*musculus*	2008/11/04	Neg		Gertler et al. 2017; Pelz et al. 2010

Mu09/0356	Baden-Wuerttemberg	Stuttgart	70173	Muridae	*Mus*	*musculus*	2008/11/13	Neg		Gertler et al. 2017; Pelz et al. 2010
Mu09/0357	Baden-Wuerttemberg	Stuttgart	70173	Muridae	*Mus*	*musculus*	2008/11/13	Neg		Gertler et al. 2017; Pelz et al. 2010
Mu09/0358	Baden-Wuerttemberg	Stuttgart	70173	Muridae	*Mus*	*musculus*	2008/11/14	Neg		Gertler et al. 2017; Pelz et al. 2010
Mu09/0359	Baden-Wuerttemberg	Stuttgart	70173	Muridae	*Mus*	*musculus*	2008/11/14	Neg		Gertler et al. 2017; Pelz et al. 2010
Mu09/0360	Baden-Wuerttemberg	Stuttgart	70173	Muridae	*Mus*	*musculus*	2008/11/14	Neg		Gertler et al. 2017; Pelz et al. 2010
Mu09/0361	Baden-Wuerttemberg	Stuttgart	70173	Muridae	*Mus*	*musculus*	2008/11/15	Neg		Gertler et al. 2017; Pelz et al. 2010
Mu09/0362	Baden-Wuerttemberg	Stuttgart	70173	Muridae	*Mus*	*musculus*	2008/11/15	Neg		Gertler et al. 2017; Pelz et al. 2010
Mu09/0363	Baden-Wuerttemberg	Stuttgart	70173	Muridae	*Mus*	*musculus*	2008/11/15	Neg		Gertler et al. 2017; Pelz et al. 2010
Mu09/0364	Baden-Wuerttemberg	Stuttgart	70173	Muridae	*Mus*	*musculus*	2008/11/16	Neg		Gertler et al. 2017; Pelz et al. 2010
Mu09/0365	Baden-Wuerttemberg	Stuttgart	70173	Muridae	*Mus*	*musculus*	2008/11/16	Neg		Gertler et al. 2017; Pelz et al. 2010
Mu09/0370	Baden-Wuerttemberg	Stuttgart	70173	Muridae	*Mus*	*musculus*	2008/11/17	Neg		Gertler et al. 2017; Pelz et al. 2010
Mu09/0371	Baden-Wuerttemberg	Stuttgart	70173	Muridae	*Mus*	*musculus*	2008/11/20	Neg		Gertler et al. 2017; Pelz et al. 2010
Mu09/0372	Baden-Wuerttemberg	Stuttgart	70173	Muridae	*Mus*	*musculus*	2008/11/20	Neg		Gertler et al. 2017; Pelz et al. 2010
Mu09/0373	Baden-Wuerttemberg	Stuttgart	70173	Muridae	*Mus*	*musculus*	2008/11/18	Neg		Gertler et al. 2017; Pelz et al. 2010
Mu09/0381	Baden-Wuerttemberg	Stuttgart	70173	Muridae	*Mus*	*musculus*	2008/12/12	Neg		Gertler et al. 2017; Pelz et al. 2010
Mu09/0382	Baden-Wuerttemberg	Stuttgart	70173	Muridae	*Mus*	*musculus*	2008/12/12	Neg		Gertler et al. 2017; Pelz et al. 2010
Mu09/0383	Baden-Wuerttemberg	Stuttgart	70173	Muridae	*Mus*	*musculus*	2008/12/12	Neg		Gertler et al. 2017; Pelz et al. 2010
Mu09/0384	Baden-Wuerttemberg	Stuttgart	70173	Muridae	*Mus*	*musculus*	2008/12/12	Neg		Gertler et al. 2017; Pelz et al. 2010
Mu09/0386	Baden-Wuerttemberg	Stuttgart	70173	Muridae	*Mus*	*musculus*	2008/12/12	Neg		Gertler et al. 2017; Pelz et al. 2010
Mu09/0387	Baden-Wuerttemberg	Stuttgart	70173	Muridae	*Mus*	*musculus*	2008/12/12	Neg		Gertler et al. 2017; Pelz et al. 2010
Mu09/0388	Baden-Wuerttemberg	Stuttgart	70173	Muridae	*Mus*	*musculus*	2008/12/12	Neg		Gertler et al. 2017; Pelz et al. 2010
Mu09/0390	Baden-Wuerttemberg	Stuttgart	70173	Muridae	*Mus*	*musculus*	2009/01/05	Neg		Gertler et al. 2017; Pelz et al. 2010
Mu09/0394	Baden-Wuerttemberg	Stuttgart	70173	Muridae	*Mus*	*musculus*	2009/02/03	Neg		Gertler et al. 2017; Pelz et al. 2010
Mu09/0396	Baden-Wuerttemberg	Stuttgart	70173	Muridae	*Mus*	*musculus*	2009/02/05	Neg		Gertler et al. 2017; Pelz et al. 2010
Mu09/0397	Baden-Wuerttemberg	Stuttgart	70173	Muridae	*Mus*	*musculus*	2009/02/05	Neg		Gertler et al. 2017; Pelz et al. 2010
Mu09/0398	Baden-Wuerttemberg	Stuttgart	70173	Muridae	*Mus*	*musculus*	2009/02/05	Neg		Gertler et al. 2017; Pelz et al. 2010
Mu09/0399	Baden-Wuerttemberg	Stuttgart	70173	Muridae	*Mus*	*musculus*	2009/02/05	Neg		Gertler et al. 2017; Pelz et al. 2010
Mu09/0400	Baden-Wuerttemberg	Stuttgart	70173	Muridae	*Mus*	*musculus*	2009/02/05	Neg		Gertler et al. 2017; Pelz et al. 2010
Mu09/0401	Baden-Wuerttemberg	Stuttgart	70173	Muridae	*Mus*	*musculus*	2009/02/05	Neg		Gertler et al. 2017; Pelz et al. 2010
Mu09/0402	Baden-Wuerttemberg	Stuttgart	70173	Muridae	*Mus*	*musculus*	2009/02/05	Neg		Gertler et al. 2017; Pelz et al. 2010
Mu09/0403	Baden-Wuerttemberg	Stuttgart	70173	Muridae	*Mus*	*musculus*	2009/02/05	Neg		Gertler et al. 2017; Pelz et al. 2010

Mu09/0404	Baden-Wuerttemberg	Stuttgart	70173	Muridae	*Mus*	*musculus*	2009/02/05	Neg		Gertler et al. 2017; Pelz et al. 2010
Mu09/0405	Baden-Wuerttemberg	Stuttgart	70173	Muridae	*Mus*	*musculus*	2009/01/28	Neg		Gertler et al. 2017; Pelz et al. 2010
Mu09/0406	Baden-Wuerttemberg	Stuttgart	70173	Muridae	*Mus*	*musculus*	2009/02/06	Neg		Gertler et al. 2017; Pelz et al. 2010
Mu09/0407	Baden-Wuerttemberg	Stuttgart	70173	Muridae	*Mus*	*musculus*	2009/01/28	Neg		Gertler et al. 2017; Pelz et al. 2010
Mu09/0413	Baden-Wuerttemberg	Stuttgart	70173	Muridae	*Mus*	*musculus*	2008/11/25	Neg		Gertler et al. 2017; Pelz et al. 2010
Mu09/0414	Baden-Wuerttemberg	Stuttgart	70173	Muridae	*Mus*	*musculus*	2008/11/25	Neg		Gertler et al. 2017; Pelz et al. 2010
Mu09/0415	Baden-Wuerttemberg	Stuttgart	70173	Muridae	*Mus*	*musculus*	2008/11/25	Neg		Gertler et al. 2017; Pelz et al. 2010
Mu09/0416	Baden-Wuerttemberg	Stuttgart	70173	Muridae	*Mus*	*musculus*	2008/11/25	Neg		Gertler et al. 2017; Pelz et al. 2010
Mu09/0419	Baden-Wuerttemberg	Stuttgart	70173	Muridae	*Mus*	*musculus*	2008/11/21	Neg		Gertler et al. 2017; Pelz et al. 2010
Mu09/0420	Baden-Wuerttemberg	Stuttgart	70173	Muridae	*Mus*	*musculus*	2008/11/21	Neg		Gertler et al. 2017; Pelz et al. 2010
Mu09/0421	Baden-Wuerttemberg	Stuttgart	70173	Muridae	*Mus*	*musculus*	2008/11/21	Neg		Gertler et al. 2017; Pelz et al. 2010
Mu09/0423	Baden-Wuerttemberg	Stuttgart	70173	Muridae	*Mus*	*musculus*	2008/11/22	Neg		Gertler et al. 2017; Pelz et al. 2010
Mu09/0424	Baden-Wuerttemberg	Stuttgart	70173	Muridae	*Mus*	*musculus*	2008/11/22	Neg		Gertler et al. 2017; Pelz et al. 2010
Mu09/0425	Baden-Wuerttemberg	Stuttgart	70173	Muridae	*Mus*	*musculus*	2008/11/22	Neg		Gertler et al. 2017; Pelz et al. 2010
Mu09/0438	Baden-Wuerttemberg	Stuttgart	70173	Muridae	*Mus*	*musculus*	2008/12/04	Neg		Gertler et al. 2017; Pelz et al. 2010
Mu09/0439	Baden-Wuerttemberg	Stuttgart	70173	Muridae	*Mus*	*musculus*	2008/12/04	Neg		Gertler et al. 2017; Pelz et al. 2010
Mu09/0441	Baden-Wuerttemberg	Stuttgart	70173	Muridae	*Mus*	*musculus*	2008/12/08	Neg		Gertler et al. 2017; Pelz et al. 2010
Mu09/0446	Baden-Wuerttemberg	Stuttgart	70173	Muridae	*Mus*	*musculus*	2008/12/09	Neg		Gertler et al. 2017; Pelz et al. 2010
Mu09/0447	Baden-Wuerttemberg	Stuttgart	70173	Muridae	*Mus*	*musculus*	2008/12/09	Neg		Gertler et al. 2017; Pelz et al. 2010
Mu09/0448	Baden-Wuerttemberg	Stuttgart	70173	Muridae	*Mus*	*musculus*	2008/12/09	Neg		Gertler et al. 2017; Pelz et al. 2010
Mu09/0449	Baden-Wuerttemberg	Stuttgart	70173	Muridae	*Mus*	*musculus*	2008/12/11	Neg		Gertler et al. 2017; Pelz et al. 2010
Mu09/0451	Baden-Wuerttemberg	Stuttgart	70173	Muridae	*Mus*	*musculus*	2008/07/08	Neg		Gertler et al. 2017; Pelz et al. 2010
Mu09/0452	Baden-Wuerttemberg	Stuttgart	70173	Muridae	*Mus*	*musculus*	2008/07/08	Neg		Gertler et al. 2017; Pelz et al. 2010
Mu09/0453	Baden-Wuerttemberg	Stuttgart	70173	Muridae	*Mus*	*musculus*	2008/07/08	Neg		Gertler et al. 2017; Pelz et al. 2010
Mu09/0454	Baden-Wuerttemberg	Stuttgart	70173	Muridae	*Mus*	*musculus*	2008/07/02	Neg		Gertler et al. 2017; Pelz et al. 2010
Mu09/0455	Baden-Wuerttemberg	Stuttgart	70173	Muridae	*Mus*	*musculus*	2008/07/02	Neg		Gertler et al. 2017; Pelz et al. 2010
Mu09/0456	Baden-Wuerttemberg	Stuttgart	70173	Muridae	*Mus*	*musculus*	2008/07/01	Neg		Gertler et al. 2017; Pelz et al. 2010
Mu09/0457	Baden-Wuerttemberg	Stuttgart	70173	Muridae	*Mus*	*musculus*	2008/07/01	Neg		Gertler et al. 2017; Pelz et al. 2010
Mu09/0458	Baden-Wuerttemberg	Stuttgart	70173	Muridae	*Mus*	*musculus*	2008/07/01	Neg		Gertler et al. 2017; Pelz et al. 2010
Mu09/0459	Baden-Wuerttemberg	Stuttgart	70173	Muridae	*Mus*	*musculus*	2008/07/01	Neg		Gertler et al. 2017; Pelz et al. 2010
Mu09/0460	Baden-Wuerttemberg	Stuttgart	70173	Muridae	*Mus*	*musculus*	2008/07/01	Neg		Gertler et al. 2017; Pelz et al. 2010

Mu09/0461	Baden-Wuerttemberg	Stuttgart	70173	Muridae	*Mus*	*musculus*	2008/07/01	Neg		Gertler et al. 2017; Pelz et al. 2010
Mu09/0462	Baden-Wuerttemberg	Stuttgart	70173	Muridae	*Mus*	*musculus*	2008/07/01	Neg		Gertler et al. 2017; Pelz et al. 2010
Mu09/0463	Baden-Wuerttemberg	Stuttgart	70173	Muridae	*Mus*	*musculus*	2008/07/01	Neg		Gertler et al. 2017; Pelz et al. 2010
Mu09/0464	Baden-Wuerttemberg	Stuttgart	70173	Muridae	*Mus*	*musculus*	2008/07/01	Neg		Gertler et al. 2017; Pelz et al. 2010
Mu09/0465	Baden-Wuerttemberg	Stuttgart	70173	Muridae	*Mus*	*musculus*	2008/07/01	Neg		Gertler et al. 2017; Pelz et al. 2010
Mu09/0466	Baden-Wuerttemberg	Stuttgart	70173	Muridae	*Mus*	*musculus*	2008/07/01	Neg		Gertler et al. 2017; Pelz et al. 2010
Mu09/0467	Baden-Wuerttemberg	Stuttgart	70173	Muridae	*Mus*	*musculus*	2008/07/01	Neg		Gertler et al. 2017; Pelz et al. 2010
Mu09/0468	Baden-Wuerttemberg	Stuttgart	70173	Muridae	*Mus*	*musculus*	2008/07/01	Neg		Gertler et al. 2017; Pelz et al. 2010
Mu09/0469	Baden-Wuerttemberg	Stuttgart	70173	Muridae	*Mus*	*musculus*	2008/07/01	Neg		Gertler et al. 2017; Pelz et al. 2010
Mu09/0470	Baden-Wuerttemberg	Stuttgart	70173	Muridae	*Mus*	*musculus*	2008/07/01	Neg		Gertler et al. 2017; Pelz et al. 2010
Mu09/0471	Baden-Wuerttemberg	Stuttgart	70173	Muridae	*Mus*	*musculus*	2008/07/01	Neg		Gertler et al. 2017; Pelz et al. 2010
Mu09/0472	Baden-Wuerttemberg	Stuttgart	70173	Muridae	*Mus*	*musculus*	2008/07/01	Neg		Gertler et al. 2017; Pelz et al. 2010
Mu09/0473	Baden-Wuerttemberg	Stuttgart	70173	Muridae	*Mus*	*musculus*	2008/07/01	Neg		Gertler et al. 2017; Pelz et al. 2010
Mu09/0474	Baden-Wuerttemberg	Stuttgart	70173	Muridae	*Mus*	*musculus*	2008/07/01	Neg		Gertler et al. 2017; Pelz et al. 2010
Mu09/0475	Baden-Wuerttemberg	Stuttgart	70173	Muridae	*Mus*	*musculus*	2008/07/01	Neg		Gertler et al. 2017; Pelz et al. 2010
Mu09/0476	Baden-Wuerttemberg	Stuttgart	70173	Muridae	*Mus*	*musculus*	2008/07/01	Neg		Gertler et al. 2017; Pelz et al. 2010
Mu09/0477	Baden-Wuerttemberg	Stuttgart	70173	Muridae	*Mus*	*musculus*	2008/07/01	Neg		Gertler et al. 2017; Pelz et al. 2010
Mu09/0478	Baden-Wuerttemberg	Stuttgart	70173	Muridae	*Mus*	*musculus*	2008/07/01	Neg		Gertler et al. 2017; Pelz et al. 2010
Mu09/0479	Baden-Wuerttemberg	Stuttgart	70173	Muridae	*Mus*	*musculus*	2008/07/01	Neg		Gertler et al. 2017; Pelz et al. 2010
Mu09/0480	Baden-Wuerttemberg	Stuttgart	70173	Muridae	*Mus*	*musculus*	2008/07/01	Neg		Gertler et al. 2017; Pelz et al. 2010
Mu09/0481	Baden-Wuerttemberg	Stuttgart	70173	Muridae	*Mus*	*musculus*	2008/07/01	Neg		Gertler et al. 2017; Pelz et al. 2010
Mu09/0482	Baden-Wuerttemberg	Stuttgart	70173	Muridae	*Mus*	*musculus*	2008/07/01	Neg		Gertler et al. 2017; Pelz et al. 2010
Mu09/0483	Baden-Wuerttemberg	Stuttgart	70173	Muridae	*Mus*	*musculus*	2008/07/01	Neg		Gertler et al. 2017; Pelz et al. 2010
Mu09/0484	Baden-Wuerttemberg	Stuttgart	70173	Muridae	*Mus*	*musculus*	2008/07/01	Neg		Gertler et al. 2017; Pelz et al. 2010
Mu09/0485	Baden-Wuerttemberg	Stuttgart	70173	Muridae	*Mus*	*musculus*	2008/07/01	Neg		Gertler et al. 2017; Pelz et al. 2010
**Total**	**Baden-Wuerttemberg**							**1/145**		
Mu09/0195	North Rhine-Westphalia	Münster	48168	Muridae	*Mus*	*musculus*		Neg		Gabriel et al. 2024
Mu09/0205	North Rhine-Westphalia	Dortmund	44287	Muridae	*Mus*	*musculus*		Neg		Pelz et al. 2010
Mu09/0206	North Rhine-Westphalia	Dortmund	44287	Muridae	*Mus*	*musculus*		Neg		Pelz et al. 2010
Mu09/0207	North Rhine-Westphalia	Dortmund	44287	Muridae	*Mus*	*musculus*		Neg		Pelz et al. 2010
Mu09/0208	North Rhine-Westphalia	Dortmund	44287	Muridae	*Mus*	*musculus*		Pos	1:16	Pelz et al. 2010

Mu09/0209	North Rhine-Westphalia	Hamm	59065	Muridae	*Mus*	*musculus*		Neg		Pelz et al. 2010
Mu09/0210	North Rhine-Westphalia	Datteln	45711	Muridae	*Mus*	*musculus*		Neg		Pelz et al. 2010
Mu09/0211	North Rhine-Westphalia	Datteln	45711	Muridae	*Mus*	*musculus*		Neg		Pelz et al. 2010
Mu09/0212	North Rhine-Westphalia	Münster	48168	Muridae	*Mus*	*musculus*		Neg		Gabriel et al. 2024
Mu09/0213	North Rhine-Westphalia	Münster	48168	Muridae	*Mus*	*musculus*		Neg		Gabriel et al. 2024
Mu09/0214	North Rhine-Westphalia	Münster	48168	Muridae	*Mus*	*musculus*		Neg		Gabriel et al. 2024
Mu09/0215	North Rhine-Westphalia	Münster	48168	Muridae	*Mus*	*musculus*		Neg		Gabriel et al. 2024
Mu09/0216	North Rhine-Westphalia	Münster	48168	Muridae	*Mus*	*musculus*		Neg		Gabriel et al. 2024
Mu09/0217	North Rhine-Westphalia	Münster	48168	Muridae	*Mus*	*musculus*		Neg		Gabriel et al. 2024
Mu09/0218	North Rhine-Westphalia	Münster	48168	Muridae	*Mus*	*musculus*		Neg		Gabriel et al. 2024
Mu09/0219	North Rhine-Westphalia	Münster	48168	Muridae	*Mus*	*musculus*		Neg		Gabriel et al. 2024
Mu09/0220	North Rhine-Westphalia	Münster	48168	Muridae	*Mus*	*musculus*		Neg		Gabriel et al. 2024
Mu09/0221	North Rhine-Westphalia	Münster	48168	Muridae	*Mus*	*musculus*		Neg		Gabriel et al. 2024
Mu09/1091	North Rhine-Westphalia	Cologne	50735	Muridae	*Mus*	*musculus*	2008/10/22	Neg		Gertler et al. 2017; Pelz et al. 2010
Mu09/1092	North Rhine-Westphalia	Cologne	50735	Muridae	*Mus*	*musculus*	2008/10/22	Neg		Gertler et al. 2017; Pelz et al. 2010
Mu09/1093	North Rhine-Westphalia	Cologne	50735	Muridae	*Mus*	*musculus*	2008/10/22	Neg		Gertler et al. 2017; Pelz et al. 2010
Mu09/1094	North Rhine-Westphalia	Cologne	50735	Muridae	*Mus*	*musculus*	2008/10/22	Neg		Gertler et al. 2017; Pelz et al. 2010
Mu09/1096	North Rhine-Westphalia	Cologne	50735	Muridae	*Mus*	*musculus*	2008/10/22	Neg		Gertler et al. 2017; Pelz et al. 2010
Mu09/1100	North Rhine-Westphalia	Cologne	50735	Muridae	*Mus*	*musculus*	2008/10/22	Neg		Gertler et al. 2017; Pelz et al. 2010
Mu09/1101	North Rhine-Westphalia	Cologne	50735	Muridae	*Mus*	*musculus*	2008/10/22	Neg		Gertler et al. 2017; Pelz et al. 2010
Mu09/1102	North Rhine-Westphalia	Cologne	50735	Muridae	*Mus*	*musculus*	2008/10/22	Neg		Gertler et al. 2017; Pelz et al. 2010
Mu09/1103	North Rhine-Westphalia	Cologne	50735	Muridae	*Mus*	*musculus*	2008/10/22	Neg		Gertler et al. 2017; Pelz et al. 2010
Mu09/1104	North Rhine-Westphalia	Cologne	50735	Muridae	*Mus*	*musculus*	2008/10/22	Neg		Gertler et al. 2017; Pelz et al. 2010
Mu09/1105	North Rhine-Westphalia	Cologne	50735	Muridae	*Mus*	*musculus*	2008/10/22	Neg		Gertler et al. 2017; Pelz et al. 2010
Mu09/1106	North Rhine-Westphalia	Cologne	50735	Muridae	*Mus*	*musculus*	2008/10/22	Neg		Gertler et al. 2017; Pelz et al. 2010
Mu09/1107	North Rhine-Westphalia	Cologne	50735	Muridae	*Mus*	*musculus*	2008/10/22	Neg		Gertler et al. 2017; Pelz et al. 2010
Mu09/1108	North Rhine-Westphalia	Cologne	50735	Muridae	*Mus*	*musculus*	2008/10/22	Neg		Gertler et al. 2017; Pelz et al. 2010
Mu09/1109	North Rhine-Westphalia	Cologne	50735	Muridae	*Mus*	*musculus*	2008/10/22	Neg		Gertler et al. 2017; Pelz et al. 2010
Mu09/1110	North Rhine-Westphalia	Cologne	50735	Muridae	*Mus*	*musculus*	2008/10/22	Neg		Gertler et al. 2017; Pelz et al. 2010
Mu09/1111	North Rhine-Westphalia	Cologne	50735	Muridae	*Mus*	*musculus*	2008/10/22	Neg		Gertler et al. 2017; Pelz et al. 2010
Mu09/1112	North Rhine-Westphalia	Cologne	50735	Muridae	*Mus*	*musculus*	2008/10/22	Neg		Gertler et al. 2017; Pelz et al. 2010

Mu09/1113	North Rhine-Westphalia	Cologne	50735	Muridae	*Mus*	*musculus*	2008/10/22	Neg		Gertler et al. 2017; Pelz et al. 2010
Mu09/1114	North Rhine-Westphalia	Cologne	50735	Muridae	*Mus*	*musculus*	2008/10/22	Neg		Gertler et al. 2017; Pelz et al. 2010
Mu09/1115	North Rhine-Westphalia	Cologne	50735	Muridae	*Mus*	*musculus*	2008/10/22	Neg		Gertler et al. 2017; Pelz et al. 2010
Mu09/1116	North Rhine-Westphalia	Cologne	50735	Muridae	*Mus*	*musculus*	2008/10/22	Neg		Gertler et al. 2017; Pelz et al. 2010
Mu09/1121	North Rhine-Westphalia	Cologne	50735	Muridae	*Mus*	*musculus*	2008/10/22	Neg		Gertler et al. 2017; Pelz et al. 2010
Mu09/1122	North Rhine-Westphalia	Cologne	50735	Muridae	*Mus*	*musculus*	2008/10/22	Neg		Gertler et al. 2017; Pelz et al. 2010
**Total**	**North Rhine-Westphalia**							**1/42**		
Mu09/1191	Saxony-Anhalt	Magdeburg	39104	Muridae	*Mus*	*musculus*	2008/03/17	Neg		Pelz et al. 2010
Mu09/1194	Saxony-Anhalt	Magdeburg	39104	Muridae	*Mus*	*musculus*	2008/03/17	Neg		Pelz et al. 2010
Mu09/1195	Saxony-Anhalt	Magdeburg	39104	Muridae	*Mus*	*musculus*	2008/03/17	Neg		Pelz et al. 2010
Mu09/1196	Saxony-Anhalt	Magdeburg	39104	Muridae	*Mus*	*musculus*	2008/08/12	Neg		Pelz et al. 2010
Mu09/1197	Saxony-Anhalt	Magdeburg	39104	Muridae	*Mus*	*musculus*	2008/08/12	Neg		Pelz et al. 2010
Mu09/1198	Saxony-Anhalt	Magdeburg	39104	Muridae	*Mus*	*musculus*	2008/03/12	Neg		Pelz et al. 2010
Mu09/1199	Saxony-Anhalt	Magdeburg	39104	Muridae	*Mus*	*musculus*	2008/03/12	Neg		Pelz et al. 2010
Mu09/1200	Saxony-Anhalt	Magdeburg	39104	Muridae	*Mus*	*musculus*	2008/03/26	Neg		Pelz et al. 2010
Mu09/1201	Saxony-Anhalt	Magdeburg	39104	Muridae	*Mus*	*musculus*	2008/03/26	Neg		Pelz et al. 2010
Mu09/1202	Saxony-Anhalt	Magdeburg	39104	Muridae	*Mus*	*musculus*	2008/05/21	Neg		Pelz et al. 2010
Mu09/1203	Saxony-Anhalt	Magdeburg	39104	Muridae	*Mus*	*musculus*	2008/05/21	Neg		Pelz et al. 2010
Mu09/1204	Saxony-Anhalt	Magdeburg	39104	Muridae	*Mus*	*musculus*	2008/05/21	Neg		Pelz et al. 2010
Mu09/1205	Saxony-Anhalt	Magdeburg	39104	Muridae	*Mus*	*musculus*	2008/05/21	Neg		Pelz et al. 2010
Mu09/1206	Saxony-Anhalt	Magdeburg	39104	Muridae	*Mus*	*musculus*	2008/03/24	Neg		Pelz et al. 2010
Mu09/1207	Saxony-Anhalt	Magdeburg	39104	Muridae	*Mus*	*musculus*	2008/03/24	Neg		Pelz et al. 2010
Mu09/1208	Saxony-Anhalt	Magdeburg	39104	Muridae	*Mus*	*musculus*	2008/03/24	Neg		Pelz et al. 2010
Mu09/1209	Saxony-Anhalt	Magdeburg	39104	Muridae	*Mus*	*musculus*	2008/03/24	Neg		Pelz et al. 2010
Mu09/1218	Saxony-Anhalt	Magdeburg	39104	Muridae	*Mus*	*musculus*	2008/08/08	Neg		Pelz et al. 2010
Mu09/1219	Saxony-Anhalt	Magdeburg	39104	Muridae	*Mus*	*musculus*	2008/08/08	Pos	1:4	Pelz et al. 2010
Mu09/1220	Saxony-Anhalt	Magdeburg	39104	Muridae	*Mus*	*musculus*	2008/08/08	Neg		Pelz et al. 2010
Mu09/1221	Saxony-Anhalt	Magdeburg	39104	Muridae	*Mus*	*musculus*	2008/10/31	Neg		Pelz et al. 2010
Mu09/1222	Saxony-Anhalt	Magdeburg	39104	Muridae	*Mus*	*musculus*	2008/04/30	Neg		Pelz et al. 2010
Mu09/1223	Saxony-Anhalt	Magdeburg	39104	Muridae	*Mus*	*musculus*	2008/04/30	Neg		Pelz et al. 2010
Mu09/1224	Saxony-Anhalt	Magdeburg	39104	Muridae	*Mus*	*musculus*	2008/04/30	Neg		Pelz et al. 2010

Mu09/1225	Saxony-Anhalt	Magdeburg	39104	Muridae	*Mus*	*musculus*	2008/03/18	Neg		Pelz et al. 2010
Mu09/1227	Saxony-Anhalt	Magdeburg	39104	Muridae	*Mus*	*musculus*	2008/06/06	Neg		Pelz et al. 2010
Mu09/1228	Saxony-Anhalt	Magdeburg	39104	Muridae	*Mus*	*musculus*	2008/06/06	Neg		Pelz et al. 2010
Mu09/1229	Saxony-Anhalt	Magdeburg	39104	Muridae	*Mus*	*musculus*	2008/03/14	Neg		Pelz et al. 2010
Mu09/1230	Saxony-Anhalt	Magdeburg	39104	Muridae	*Mus*	*musculus*	2008/03/14	Neg		Pelz et al. 2010
Mu09/1231	Saxony-Anhalt	Magdeburg	39104	Muridae	*Mus*	*musculus*	2008/08/15	Neg		Pelz et al. 2010
Mu09/1232	Saxony-Anhalt	Magdeburg	39104	Muridae	*Mus*	*musculus*	2008/05/20	Neg		Pelz et al. 2010
Mu09/1233	Saxony-Anhalt	Magdeburg	39104	Muridae	*Mus*	*musculus*	2008/08/08	Neg		Pelz et al. 2010
Mu09/1234	Saxony-Anhalt	Magdeburg	39104	Muridae	*Mus*	*musculus*	2008/03/11	Neg		Pelz et al. 2010
Mu09/1235	Saxony-Anhalt	Magdeburg	39104	Muridae	*Mus*	*musculus*	2008/03/11	Neg		Pelz et al. 2010
Mu09/1236	Saxony-Anhalt	Magdeburg	39104	Muridae	*Mus*	*musculus*	2008/06/06	Neg		Pelz et al. 2010
Mu09/1237	Saxony-Anhalt	Magdeburg	39104	Muridae	*Mus*	*musculus*	2008/09/19	Neg		Pelz et al. 2010
Mu09/1238	Saxony-Anhalt	Magdeburg	39104	Muridae	*Mus*	*musculus*	2008/09/19	Neg		Pelz et al. 2010
Mu09/1239	Saxony-Anhalt	Magdeburg	39104	Muridae	*Mus*	*musculus*	2008/09/19	Neg		Pelz et al. 2010
Mu09/1240	Saxony-Anhalt	Magdeburg	39104	Muridae	*Mus*	*musculus*	2008/06/11	Neg		Pelz et al. 2010
Mu09/1241	Saxony-Anhalt	Magdeburg	39104	Muridae	*Mus*	*musculus*	2008/05/07	Neg		Pelz et al. 2010
Mu09/1242	Saxony-Anhalt	Magdeburg	39104	Muridae	*Mus*	*musculus*		Neg		Pelz et al. 2010
Mu09/1243	Saxony-Anhalt	Magdeburg	39104	Muridae	*Mus*	*musculus*	2008/07/23	Neg		Pelz et al. 2010
Mu09/1244	Saxony-Anhalt	Magdeburg	39104	Muridae	*Mus*	*musculus*	2008/09/23	Neg		Pelz et al. 2010
Mu09/1245	Saxony-Anhalt	Magdeburg	39104	Muridae	*Mus*	*musculus*	2008/09/23	Neg		Pelz et al. 2010
Mu09/1246	Saxony-Anhalt	Magdeburg	39104	Muridae	*Mus*	*musculus*	2008/09/23	Neg		Pelz et al. 2010
Mu09/1247	Saxony-Anhalt	Magdeburg	39104	Muridae	*Mus*	*musculus*	2008/05/01	Neg		Pelz et al. 2010
Mu09/1248	Saxony-Anhalt	Magdeburg	39104	Muridae	*Mus*	*musculus*	2008/05/01	Neg		Pelz et al. 2010
Mu09/1249	Saxony-Anhalt	Magdeburg	39104	Muridae	*Mus*	*musculus*	2008/05/01	Neg		Pelz et al. 2010
Mu09/1250	Saxony-Anhalt	Magdeburg	39104	Muridae	*Mus*	*musculus*	2008/06/14	Neg		Pelz et al. 2010
Mu09/1251	Saxony-Anhalt	Magdeburg	39104	Muridae	*Mus*	*musculus*	2008/06/14	Neg		Pelz et al. 2010
Mu09/1252	Saxony-Anhalt	Magdeburg	39104	Muridae	*Mus*	*musculus*	2008/06/14	Neg		Pelz et al. 2010
Mu09/1253	Saxony-Anhalt	Magdeburg	39104	Muridae	*Mus*	*musculus*	2008/06/14	Neg		Pelz et al. 2010
Mu09/1254	Saxony-Anhalt	Magdeburg	39104	Muridae	*Mus*	*musculus*	2008/09/20	Neg		Pelz et al. 2010
Mu09/1255	Saxony-Anhalt	Magdeburg	39104	Muridae	*Mus*	*musculus*	2008/05/21	Neg		Pelz et al. 2010
Mu09/1256	Saxony-Anhalt	Magdeburg	39104	Muridae	*Mus*	*musculus*	2008/04/05	Neg		Pelz et al. 2010

Mu09/1257	Saxony-Anhalt	Magdeburg	39104	Muridae	*Mus*	*musculus*	2008/03/22	Neg		Pelz et al. 2010
Mu09/1258	Saxony-Anhalt	Magdeburg	39104	Muridae	*Mus*	*musculus*	2008/03/22	Neg		Pelz et al. 2010
Mu09/1259	Saxony-Anhalt	Magdeburg	39104	Muridae	*Mus*	*musculus*	2008/03/22	Neg		Pelz et al. 2010
Mu09/1260	Saxony-Anhalt	Magdeburg	39104	Muridae	*Mus*	*musculus*	2008/09/20	Neg		Pelz et al. 2010
Mu09/1261	Saxony-Anhalt	Magdeburg	39104	Muridae	*Mus*	*musculus*	2008/09/20	Neg		Pelz et al. 2010
Mu09/1262	Saxony-Anhalt	Magdeburg	39104	Muridae	*Mus*	*musculus*	2008/10/07	Neg		Pelz et al. 2010
Mu09/1263	Saxony-Anhalt	Magdeburg	39104	Muridae	*Mus*	*musculus*	2008/10/07	Neg		Pelz et al. 2010
Mu09/1264	Saxony-Anhalt	Magdeburg	39104	Muridae	*Mus*	*musculus*	2008/10/07	Neg		Pelz et al. 2010
Mu09/1265	Saxony-Anhalt	Magdeburg	39104	Muridae	*Mus*	*musculus*	2008/03/16	Neg		Pelz et al. 2010
Mu09/1266	Saxony-Anhalt	Magdeburg	39104	Muridae	*Mus*	*musculus*	2008/08/14	Neg		Pelz et al. 2010
Mu09/1267	Saxony-Anhalt	Magdeburg	39104	Muridae	*Mus*	*musculus*	2008/08/14	Neg		Pelz et al. 2010
Mu09/1268	Saxony-Anhalt	Magdeburg	39104	Muridae	*Mus*	*musculus*	2008/08/14	Neg		Pelz et al. 2010
Mu09/1269	Saxony-Anhalt	Magdeburg	39104	Muridae	*Mus*	*musculus*	2008/08/14	Neg		Pelz et al. 2010
Mu09/1270	Saxony-Anhalt	Magdeburg	39104	Muridae	*Mus*	*musculus*	2008/08/14	Neg		Pelz et al. 2010
Mu09/1271	Saxony-Anhalt	Magdeburg	39104	Muridae	*Mus*	*musculus*	2008/08/14	Neg		Pelz et al. 2010
Mu09/1272	Saxony-Anhalt	Magdeburg	39104	Muridae	*Mus*	*musculus*	2008/08/13	Neg		Pelz et al. 2010
Mu09/1273	Saxony-Anhalt	Magdeburg	39104	Muridae	*Mus*	*musculus*	2008/08/13	Neg		Pelz et al. 2010
Mu09/1274	Saxony-Anhalt	Magdeburg	39104	Muridae	*Mus*	*musculus*	2008/08/13	Neg		Pelz et al. 2010
Mu09/1275	Saxony-Anhalt	Magdeburg	39104	Muridae	*Mus*	*musculus*	2008/08/13	Neg		Pelz et al. 2010
Mu09/1276	Saxony-Anhalt	Magdeburg	39104	Muridae	*Mus*	*musculus*	2008/08/13	Neg		Pelz et al. 2010
Mu09/1277	Saxony-Anhalt	Magdeburg	39104	Muridae	*Mus*	*musculus*	2008/03/29	Neg		Pelz et al. 2010
Mu09/1278	Saxony-Anhalt	Magdeburg	39104	Muridae	*Mus*	*musculus*	2008/03/31	Neg		Pelz et al. 2010
**Total**	**Saxony-Anhalt**							**1/77**		
Mu08/0696	Schleswig-Holstein	Helgoland	27498	Muridae	*Mus*	*musculus*	2008/04/19	Neg		Harr et al. 2016
Mu08/0697	Schleswig-Holstein	Helgoland	27498	Muridae	*Mus*	*musculus*	2008/04/12	Neg		Harr et al. 2016
Mu08/0698	Schleswig-Holstein	Helgoland	27498	Muridae	*Mus*	*musculus*	2008/04/16	Neg		Harr et al. 2016
Mu08/0700	Schleswig-Holstein	Helgoland	27498	Muridae	*Mus*	*musculus*	2008/04/13	Neg		Harr et al. 2016
Mu08/0701	Schleswig-Holstein	Helgoland	27498	Muridae	*Mus*	*musculus*	2008/04/12	Neg		Harr et al. 2016
Mu08/0702	Schleswig-Holstein	Helgoland	27498	Muridae	*Mus*	*musculus*	2008/04/04	Neg		Harr et al. 2016
**Total**	**Schleswig-Holstein**							**0/6**		

References

1. Pelz, H.-J.; Rost, S.; Müller, E.; Esther, A.; Ulrich, R.G.; Müller, C.R. Distribution and frequency of VKORC1 sequence variants conferring resistance to anticoagulants in *Mus musculus*. Pest Manag Sci **2012**, 68, 254–259, doi:10.1002/ps.2254.
2. Harr, B.; Karakoc, E.; Neme, R.; Teschke, M.; Pfeifle, C.; Pezer, Ž.; Babiker, H.; Linnenbrink, M.; Montero, I.; Scavetta, R.; et al. Genomic resources for wild populations of the house mouse, *Mus musculus* and its close relative *Mus spretus*. Sci. Data **2016**, 3, 160075, doi:10.1038/sdata.2016.75.
3. Gertler, C.; Schlegel, M.; Linnenbrink, M.; Hutterer, R.; König, P.; Ehlers, B.; Fischer, K.; Ryll, R.; Lewitzki, J.; Sauer, S.; et al. Indigenous house mice dominate small mammal communities in northern Afghan military bases. BMC Zool **2017**, 2, doi:10.1186/s40850-017-0024-z.
4. Gabriel, S.I.; Hughes, J.J.; Herman, J.S.; Baines, J.F.; Giménez, M.D.; Gray, M.M.; Hardouin, E.A.; Payseur, B.A.; Ryan, P.G.; Sánchez-Chardi, A.; et al. House Mice in the Atlantic Region: Genetic Signals of Their Human Transport. Genes (Basel) **2024**, 15, doi:10.3390/genes15121645.
